# Golgi-IP, a tool for multimodal analysis of Golgi molecular content

**DOI:** 10.1073/pnas.2219953120

**Published:** 2023-05-08

**Authors:** Rotimi Fasimoye, Wentao Dong, Raja S. Nirujogi, Eshaan S. Rawat, Miharu Iguchi, Kwamina Nyame, Toan K. Phung, Enrico Bagnoli, Alan R. Prescott, Dario R. Alessi, Monther Abu-Remaileh

**Affiliations:** ^a^Medical Research Council Protein Phosphorylation and Ubiquitylation Unit, School of Life Sciences, University of Dundee, Dundee DD1 5EH, United Kingdom; ^b^Aligning Science Across Parkinson’s Collaborative Research Network, Chevy Chase, MD 20815; ^c^Department of Chemical Engineering, Stanford University, Stanford, CA 94305; ^d^Department of Genetics, Stanford University, Stanford, CA 94305; ^e^The Institute for Chemistry, Engineering & Medicine for Human Health, Stanford University, Stanford, CA 94305; ^f^Department of Biochemistry, Stanford University School of Medicine, Stanford, CA 94305; ^g^Dundee Imaging Facility, School of Life Sciences, University of Dundee, Dundee DD1 5EH, United Kingdom

**Keywords:** Golgi, organelle, proteomics, metabolomics, lipidomics

## Abstract

The Golgi is a membrane-bound organelle that is essential for protein and lipid biosynthesis. It represents a central trafficking hub that sorts proteins and lipids to various destinations or for secretion from the cell. The Golgi has emerged as a docking platform for cellular signaling pathways including LRRK2 kinase whose deregulation leads to Parkinson disease. Golgi dysfunction is associated with a broad spectrum of diseases including cancer, neurodegeneration, and cardiovascular diseases. To allow the study of the Golgi at high resolution, we report a rapid Golgi immunoprecipitation technique (Golgi-IP) to isolate intact Golgi mini-stacks for subsequent analysis of their content. By fusing the Golgi-resident protein TMEM115 to three tandem HA epitopes (GolgiTAG), we purified the Golgi using Golgi-IP with minimal contamination from other compartments. We then established an analysis pipeline using liquid chromatography coupled with mass spectrometry to characterize the human Golgi proteome, metabolome, and lipidome. Subcellular proteomics confirmed known Golgi proteins and identified proteins not previously associated with the Golgi. Metabolite profiling established the human Golgi metabolome and revealed the enrichment of uridine-diphosphate (UDP) sugars and their derivatives, which is consistent with their roles in protein and lipid glycosylation. Furthermore, targeted metabolomics validated SLC35A2 as the subcellular transporter for UDP-hexose. Finally, lipidomics analysis showed that phospholipids including phosphatidylcholine, phosphatidylinositol, and phosphatidylserine are the most abundant Golgi lipids and that glycosphingolipids are enriched in this compartment. Altogether, our work establishes a comprehensive molecular map of the human Golgi and provides a powerful method to study the Golgi with high precision in health and disease.

## Significance

The Golgi is central to protein and lipid processing. It senses and responds to diverse cell states to allow trafficking of macromolecules based on cellular demands. Traditional techniques for purifying the Golgi shaped our understanding of its functions; however, such methods are too slow to preserve the labile Golgi metabolome and transient protein interactions. Here, we overcome this issue through the development of a method for the rapid capture of intact Golgi from human cells via Golgi immunoprecipitation (Golgi-IP). Using high-resolution mass spectrometry, we demonstrate that our approach allows the unbiased characterization of the Golgi proteome, metabolome, and lipidome. We believe that the Golgi-IP will be useful for the study of the Golgi in health and disease states.

The Golgi is a membrane-bound organelle central to protein and lipid processing, sorting, and secretion. After synthesis in the endoplasmic reticulum (ER), secreted and organellar proteins undergo post-translational modifications in the Golgi including glycosylation, phosphorylation, and tyrosine sulfation, processes that are essential for structural diversification, functional maturation, and signaling (1, 2). Golgi dysfunction is associated with a multitude of human disorders including cancer, neurodegeneration, and cardiovascular diseases (3–5).

To perform its functions, the Golgi relies on an adequate supply of metabolites required for modifying proteins and lipids (6, 7). Furthermore, the emerging dynamic nature of the Golgi suggests that it is capable of sensing and responding to diverse cell states to allow trafficking of macromolecules based on cellular demands (8, 9).

While traditional techniques for purifying the Golgi from mammalian cells, such as density-based centrifugation, helped shape our current understanding of its cellular roles, such methods are too slow to preserve what is likely a labile Golgi metabolome and transient protein interactions that regulate its functions. To overcome this, we used insights from our work and others to purify cellular organelles (10–18), to develop a method for the rapid capture of intact Golgi from human cells using organelle-specific immunoprecipitation (Golgi-IP).

Using our Golgi-IP method, we present a comprehensive characterization of the human Golgi proteome, metabolome, and lipidome. Golgi-IP successfully enriched previously reported Golgi proteins while revealing proteins not previously associated with this organelle. Furthermore, uridine-diphosphate (UDP) sugars and their derivatives as well as phospholipids were enriched in our untargeted metabolomics and lipidomics analyses, respectively. Finally, we used the Golgi-IP method to show that loss of the UDP-galactose transporter SLC35A2 (19, 20) leads to depletion of UDP-hexose specifically in the Golgi while its levels in the whole cell remain unchanged.

These data show that the Golgi-IP method coupled with modern omics technologies provides a powerful approach to study, in an unbiased manner, the molecular content of the Golgi and to uncover its regulation and function in health and disease states.

## Results

### Development and Validation of the Golgi-IP Method.

**Fig. 1.**
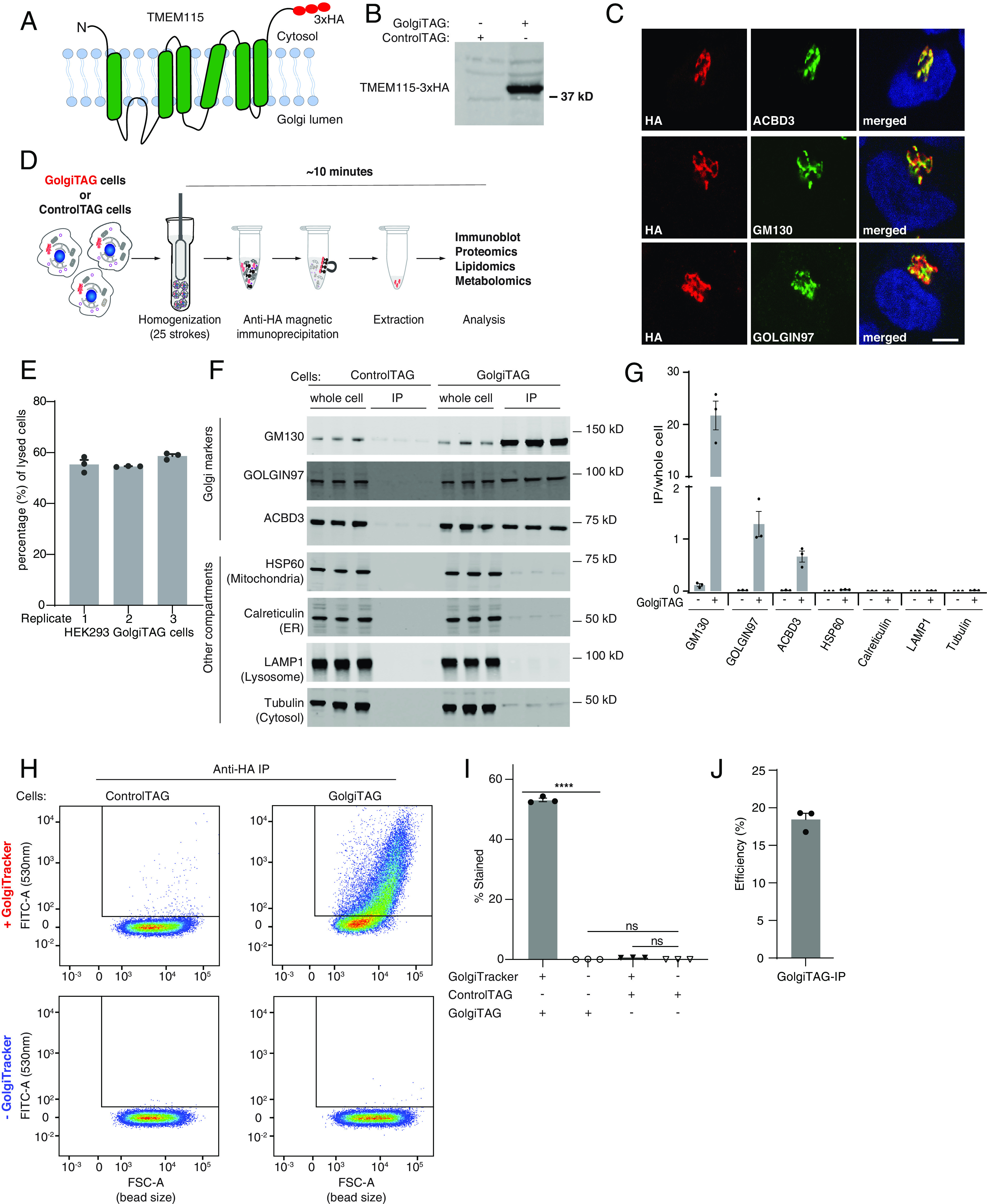
Development of the Golgi immunoprecipitation (Golgi-IP) method. (*A*) A depiction of the domain structure of the Golgi transmembrane protein TMEM115 fused to 3×-HA tag. (*B*) Immunoblot analysis of HEK293 cells stably expressing the TMEM115-3×-HA tag (GolgiTAG) using the HA antibody. (*C*) Immunofluorescence analyses confirm the Golgi localization of the GolgiTAG. HA in red and Golgi markers in green. The *Right* panel displays a merged green and red channel. (Scale bar is 1 µm). (*D*) Representation of the workflow of the Golgi-IP method (see *Methods* section and protocols.io (10.17504/protocols.io.6qpvrdjrogmk/v1 for details). (*E*) Efficiency of homogenization with glass Douncer. GolgiTAG cells from a 15-cm diameter dish were resuspended in 1 mL KPBS and the number of cells was determined using a cell counter. The cells were then homogenized in glass Douncer with 25 upward/downward strokes and the total number of the remaining cells was counted. To determine the number of lysed cells, the number of cells after homogenization was subtracted from the number of cells before homogenization. The percentage of lysed cells relative to the total number of cells before homogenization is shown. Data are presented as mean ± SEM for 3 technical replicates. (*F*) Immunoblot analyses confirm that Golgi-IP enriches for Golgi. Whole-cell lysates (2 µg) as well as the resuspended immunoprecipitates (IPs) (2 µg) were subjected to immunoblotting with the indicated compartment-specific antibodies. Immunoblotting data are shown from 3 independent biological replicates (Golgi-IP for each replicate was done on different days). (*G*) Quantitative immunoblotting analysis. The graph represents ratios of IP/whole-cell lysate (mean ± SEM). (*H*) Golgi-IP enriches for intact Golgi. Control HEK293 and HEK293 cells stably expressing the GolgiTAG were stained with and without 5 µM BODIPY^TM^ FL C5-Ceramide (GolgiTracker) for 30 min at 37 °C. Cells were lysed and subjected to Golgi-IP as in *F* except that the IP fraction was resuspended in KPBS and subjected to flow cytometry analysis using the FITC 530 nm channel. The beads bound to Golgi were first gated and selected as described in *SI Appendix*, Fig. S1*A*. The selected beads were then analyzed based on emission intensity in the FITC 530 nm (*Y* axis) and bead size (forward side scatter FSC, *X* axis). (*I*) Quantitation of the percentage of beads stained with the GolgiTracker of 3 replicates. Data are presented as mean ± SEM (one-way ANOVA). (*J*) Golgi-IP efficiency by quantitative immunoblotting. 0.5% whole-cell lysate and 5% total immunoprecipitate from three independent experiments were subjected to quantitative immunoblotting with anti-GM130 (*cis*-Golgi protein) antibody.

Rapid purification of cellular organelles including mitochondria (12, 14, 15, 17), lysosomes (10, 11), peroxisomes (16), and early-endosomes (13) have allowed the analysis of their molecular content at unprecedented resolution. To develop an analogous approach to isolate intact Golgi, we generated a fusion protein comprised of the Golgi-specific transmembrane protein, TMEM115 (21, 22), whose N- and C-termini face the cytoplasm, fused to three tandem HA-epitopes at its C terminus (TMEM115-3×HA; referred to as GolgiTAG) (Fig. 1*A*). Although the exact function of TMEM115 is not known, it interacts with the conserved oligomeric Golgi complex that is essential for the localization of Golgi-resident enzymes and the tethering of transport vesicles to the Golgi (23). Endogenous TMEM115 has an estimated copy number of ~80,000 in HEK293 cells (*Methods*).

To immunopurify the Golgi from human cells, we generated human embryonic kidney cells (HEK293) stably expressing the GolgiTAG (Fig. 1 *A* and *B*). As a control, we generated HEK293 cells stably expressing the empty backbone vector used to express the GolgiTAG, which retains the region encoding for the puromycin resistance and the 3×HA peptide (ControlTAG). Immunofluorescence microscopy confirmed that the GolgiTAG colocalizes with markers of the Golgi cisternae (ACBD3), *cis*-Golgi network (GM130/GOLGA2), and the *trans*-Golgi network (Golgin97/GOLGA1) (Fig. 1*C*). Of importance, the expression of the tag did not cause Golgi fragmentation (Fig. 1*C*), which is commonly observed upon depletion or overexpression of other Golgi proteins (24). After validating the localization of the GolgiTAG, we next homogenized GolgiTAG and ControlTAG HEK293 cells in isotonic potassium-supplemented phosphate-buffer saline (KPBS) and performed an anti-HA immunoprecipitation of the Golgi using magnetic beads (Fig. 1*D* and *Methods*). We estimated that approximately 55% of HEK293 cells were homogenized after 25 strokes in a Glass Dounce homogenizer (Fig. 1*E*). To minimize the leakage of metabolites and the degradation of labile content, the Golgi-IP was optimized to take ~10 min to isolate Golgi, starting with live cells. Immunoblot analyses of the bead-bound fraction revealed that GM130, GOLGIN97, and ACBD3 Golgi proteins were enriched in the immunoprecipitates (IPs) from GolgiTAG cells compared to markers for ER (calreticulin), mitochondria (HSP60), cytosol (tubulin), and lysosomes (LAMP1), which were depleted (Fig. 1 *F* and *G*). No enrichment was observed in IPs from ControlTAG cells (Fig. 1 *F* and *G*). Similar results were obtained in 3 independent experiments. Furthermore, to test whether Golgi-IP preserves the metabolite content of purified organelles, cells were pretreated with the Golgi-specific ceramide-based fluorescent dye (GolgiTracker, see *Methods*) before Golgi-IP. Indeed, IPs from GolgiTAG cells were enriched for GolgiTracker, while no signal was observed in those from ControlTAG cells (Fig. 1 *H* and *I* and *SI Appendix*, Fig. S1*A*). We determined the efficiency of the Golgi-IP to be around 18%, by quantifying immunoprecipitation of the Golgi protein GM130 (Fig. 1*J* and *SI Appendix*, Fig. S1*B*). Finally, representative transmission electron micrographs of the bound fraction showed that our preparation contained a mixture of cisternae-like structures likely from disrupted ministacks and some vesicle-like structures, that are possibly Golgi ministacks and/or vesicles derived from the Golgi. An intact Golgi ministack is also shown (*SI Appendix*, Fig. S1*C*).

### Golgi-IP Proteomics Defines the Golgi Proteome and Reveals Proteins not Previously Associated with the Golgi.

**Fig. 2.**
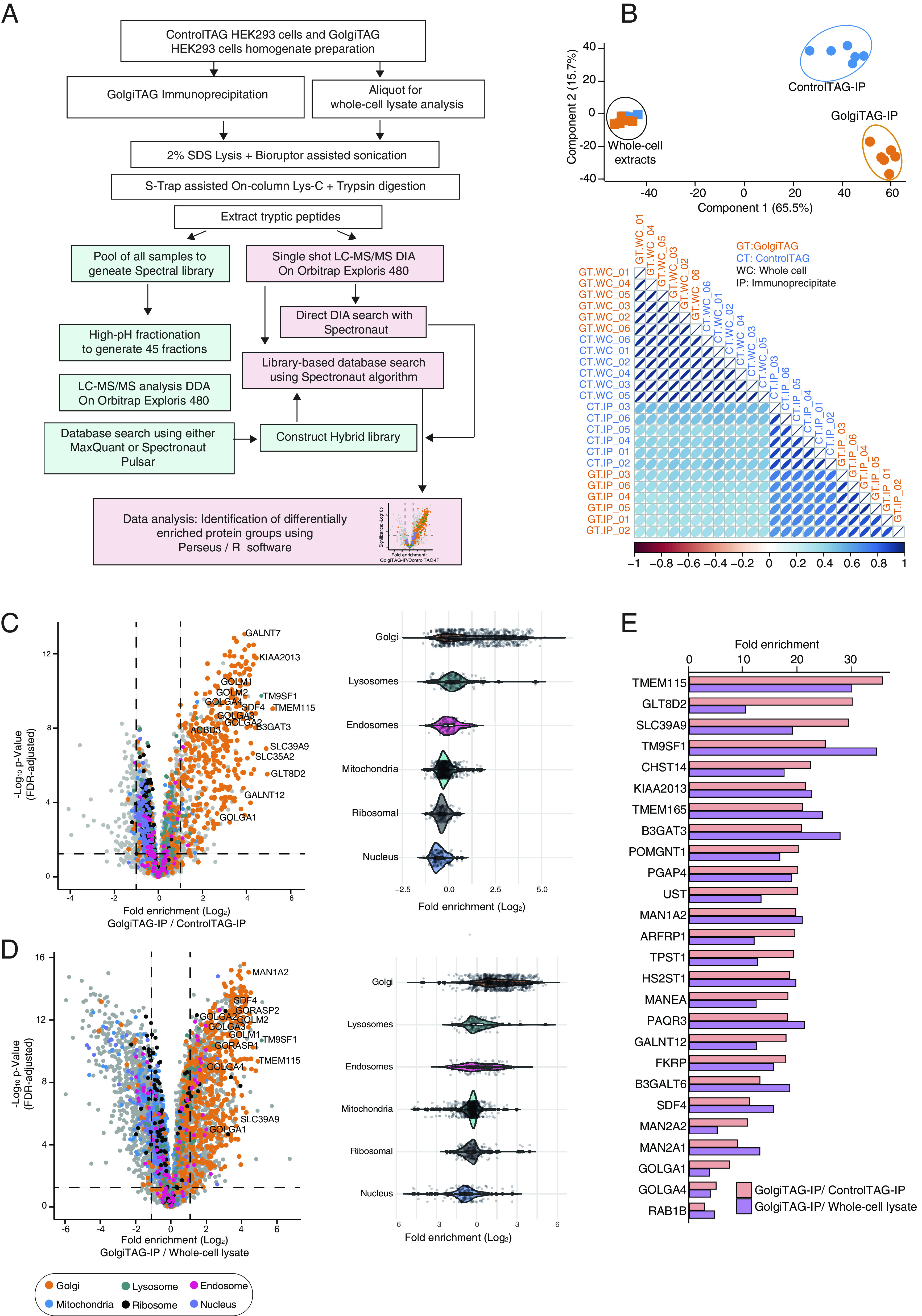
Quantitative proteomic analysis of Golgi derived from HEK293 cells. (*A*) Depiction of the workflow of the data-independent acquisition (DIA)-based mass spectrometry analysis of whole-cell lysates and HA immunoprecipitates (IPs) from control and GolgiTAG expressing HEK293 cells n = 6. (*B*) Principal component analysis of DIA mass spectrometry data of whole-cell lysates (squares) and IPs (circle) from the control (blue) and GolgiTAG expressing HEK293 cells (orange) (*Upper*). Lower panel presents a global Pearson’s correlation of proteomics data of all samples. The control immunoblots for this experiment are presented in *SI Appendix*, Fig. S2*A*. (*C*) Volcano plot of the fold enrichment of proteins between IPs from GolgiTAG and control HEK293 cells (n = 6 *P*-value adjusted for 1% permutation-based FDR correction, s0 = 0.1) (*Left*). Selected well-studied Golgi proteins are denoted with gene names. The orange dots indicate known Golgi-annotated proteins curated from databases described in Dataset S3. Proteins associated with the lysosome, endosome, mitochondria, ribosome, and nucleus are indicated with the colors represented below the volcano plot. The adjacent violin plots (*Right*) represent the relative fold enrichment of the proteins associated with indicated organelles. (*D*) As in (*C*) except that the fold enrichment in protein abundances was calculated by comparing the IP fraction and the whole-cell lysate of GolgiTAG cells. (*E*) Bar graph presentation of the relative fold enrichment of top 26 proteins enriched in the IP fraction from GolgiTAG cells compared to those from ControlTAG cells (orange) or to whole-cell lysate from GolgiTAG cells (purple).

To determine the proteomic landscape of the human Golgi, we used high-resolution data-independent acquisition (DIA) liquid chromatography/tandem mass spectrometry (LC/MS-MS), to analyze the protein content of whole-cell and IP lysates derived from GolgiTAG- and ControlTAG cells, n = 6 (Fig. 2 *A* and *B*, *SI Appendix*, Fig. S2 *A*–*F*, Datasets S1 and S2, and *Methods*). As expected, principal component and correlation matrix analyses revealed the distinct proteomic profile of IPs derived from GolgiTAG cells when compared to those derived from ControlTAG cells or the whole-cell fractions of either line (Fig. 2*B*). Note that no major change in global protein abundances was observed between lysates collected from GolgiTAG and ControlTAG whole cells (*SI Appendix*, Fig. S3*A* and Dataset S1); thus, the expression of TMEM115-3×HA has no detrimental effects on the cellular proteome including on proteins involved in the unfolded protein response (UPR), C/EBP Homologous Protein (CHOP), and integrated stress response (ISR) responses (*SI Appendix*, Fig. S3*A* and Dataset S1). To further analyze this dataset, we first curated a comprehensive list of 1,363 proteins that were previously annotated as Golgi proteins in the compartment database or recently published subcellular organelle proteomic studies (25–30) (Dataset S3). In this curated list, we included the estimated protein copy numbers for each protein in HEK293 whole-cell extracts (Dataset S3). These range from high abundance ~10 million copies per cell (TAGLN2, PARK7, CALU, and RAB1A) to low abundance <1,000 copies per cell (MACF1, COPA, and TMED5) (Dataset S3 and *SI Appendix*, Fig. S3*B*). Of the ~7,000 proteins detected in our dataset, 527 were significantly at least twice as abundant in the isolated GolgiTAG IP compared to ControlTAG IP (adjusted *P*-value with 1% permutation-based FDR). A total of 360 out of the 527 proteins were previously reported as Golgi-associated proteins in our curated list, whereas the remaining 167 proteins were not (Fig. 2*C* and Dataset S1). Furthermore, 463 proteins were deemed Golgi-associated proteins when enrichment was calculated by comparing protein abundance in GolgiTAG IP to that of the whole-cell (at least twofold enrichment, adjusted *P*-value with 1% permutation-based false discovery rate (FDR) (Fig. 2*D* and Dataset S1). Lastly, out of 115 proteins predicted to be secretory proteins due to the presence of signal peptides (31), our Golgi-IP protocol enriched for 83 of these proteins, while the remaining 32 proteins were identified but not enriched (Dataset S1). To further analyze this dataset, we also compared the enrichment of verified lysosomal, endosomal, mitochondrial, ribosomal, and nuclear proteins to Golgi proteins in GolgiTAG IP versus whole-cell lysate (Fig. 2*C*) or ControlTAG IP (Fig. 2*D*). Golgi proteins are more enriched in this dataset than the proteins from other compartments.

Among the identified proteins are the well-characterized Golgi proteins ACBD3 (5.3-fold) GM130/GOLGA2 (7.8-fold), Golgin-97/GOLGA1 (7.5-fold), B3GALT6 (13.1-fold), the Golgi luminal protein SDF4 (11.2-fold), GOLGA4 (4.9-fold), Rab29 (1.8-fold), Rab1B (2.7-fold), MAN2A1 (8.9-fold), and MAN2A2 (10.9-fold) (Fig. 2*E * and Dataset S1). Furthermore, we analyzed the GolgiTAG isolated proteins (GolgiTAG IP/ControlTAG IP and GolgiTAG IP/whole-cell) for machineries required for Golgi functions including glycosylation, protein phosphorylation and dephosphorylation, and protein ubiquitylation (*SI Appendix*, Fig. S3 *C* and *D*). Interestingly, FAM20C (5.7-fold), the Golgi Casein kinase implicated to play a major role in secretory phosphoproteome, was also enriched (32–35).

As expected, the vast majority of the glycosylation-related proteins are indeed detected and enriched in the GolgiTAG IPs (Dataset S1). The Golgi houses a large number of G proteins and our data showed that known G proteins such as ARFRP1 (19.7-fold) and RAB33B (4.3-fold) are enriched (*SI Appendix*, Fig. S3*E*).

**Fig. 3.**
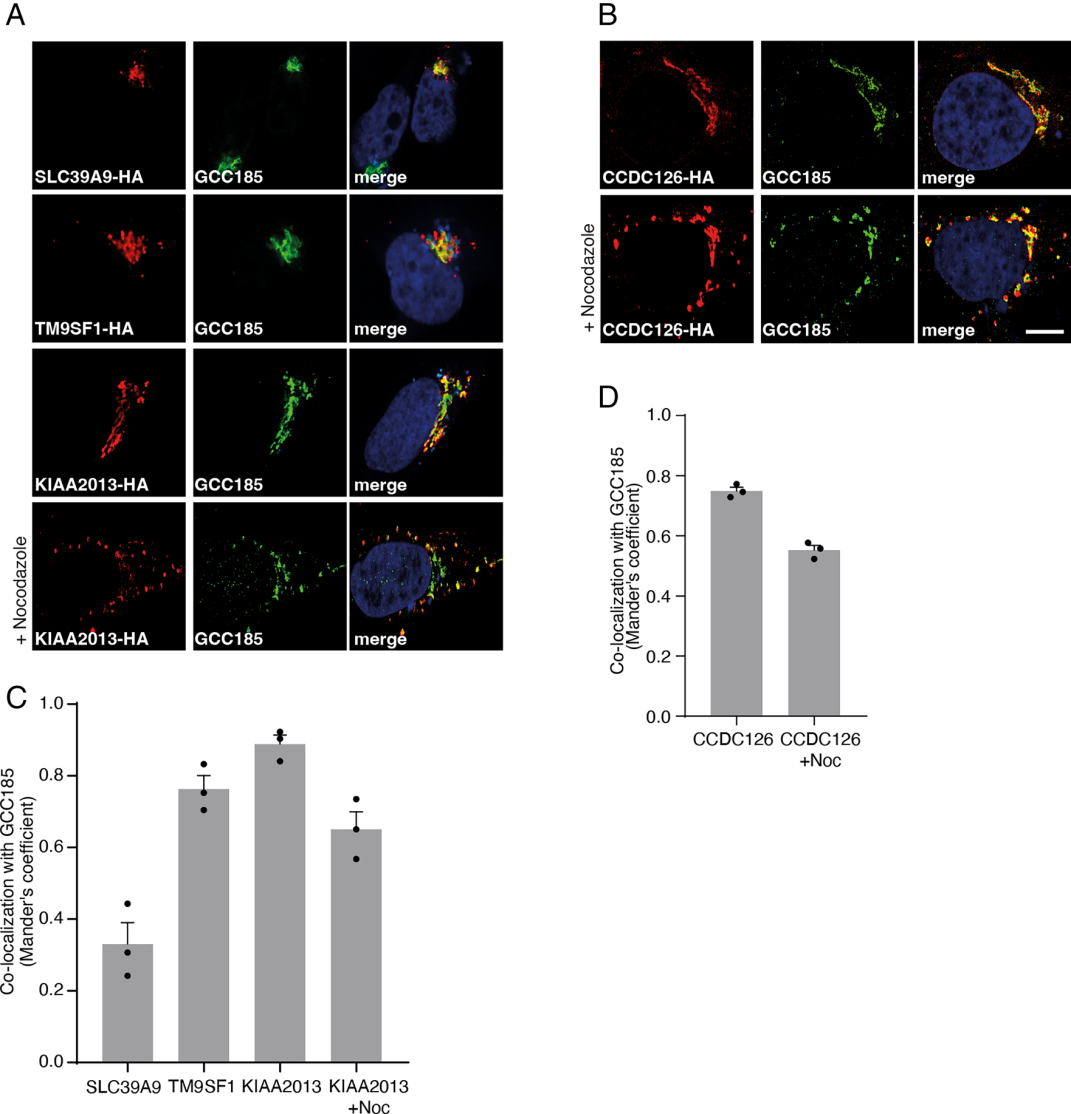
Immunofluorescence of proteins enriched in the Golgi. (*A* and *B*) Validation of Golgi localization of proteins found in the GolgiTAG IP. HeLa cells were transiently transfected with plasmids encoding the indicated proteins fused to a C-terminal HA tag. Cells were then fixed and stained with rat anti-HA (red, *Left*) and rabbit anti-GCC185 (green, *Middle*). The *Right* panel displays the merged red and green channel. Nuclei were stained with DAPI (blue). For nocodazole-treated samples, cells were treated with ± 30 µM nocodazole for 1 h at 37 °C 24 h post transfection. (Scale bar is 1 µm). (*C* and *D*) Colocalization of HA-tagged protein and GCC185 was quantified using Mander’s coefficient and data (from three biological replicates) are presented as mean ± SEM after automatic thresholding. For each condition, at least 15 cells were analyzed (i.e., at least 5 cells per biological replicate).

From the list of 360 proteins identified as enriched in the Golgi, we performed immunofluorescence analysis of the localization of SLC39A9 (29.7-fold) (36), TM9SF1 (transmembrane9 superfamily member 1) (25.4-fold) (37), and KIAA2013 (21.4-fold) (27–29) and confirmed previous reports that these proteins colocalize with the *trans*-Golgi network protein, GCC185 (38, 39) (Fig. 3 *A*–*C*). As there was no previous immunofluorescence data for KIAA2013, we treated cells with nocodazole to induce microtubule depolymerization, a well-established approach to verify Golgi compartment localization (39, 40). We found that nocodazole treatment does not disrupt the colocalization of KIAA2013 with GCC185 (Fig. 3 *B*–*D*), further confirming KIAA2013 as a Golgi-resident protein.

To study proteins not previously associated with the Golgi, we turned to the list of 167 proteins that show significant enrichment (≥twofold and adjusted *P*-value with 1% FDR) in our GolgiTAG IPs but have not been reported as potential Golgi proteins (Datasets S1 and S3). By manually searching the Uniprot database, we identified 42 proteins to be annotated as putative ER proteins and 55 proteins that are associated with endosomes, lysosomes, and centrioles. Of the remaining 70 proteins, we tested a few for their potential Golgi localization using over expression coupled with immunofluorescence analyses; these included DIPK1A (17.5-fold), C5orf22 (16.7-fold), CCDC126 (11.2-fold), TMEM219 (11.0-fold), and ABHD13 (10.2-fold). Of these, we observed a strong colocalization of CCDC126 with GCC185 that was not impacted by nocodazole treatment (Fig. 3 *B*–*D*). In contrast, we did not observe strong colocalization of DIPK1A, C5orf22, TMEM219, and ABHD13 with GCC185 (*SI Appendix*, Fig. S4).

### The Metabolic Landscape of the Human Golgi.

**Fig. 4.**
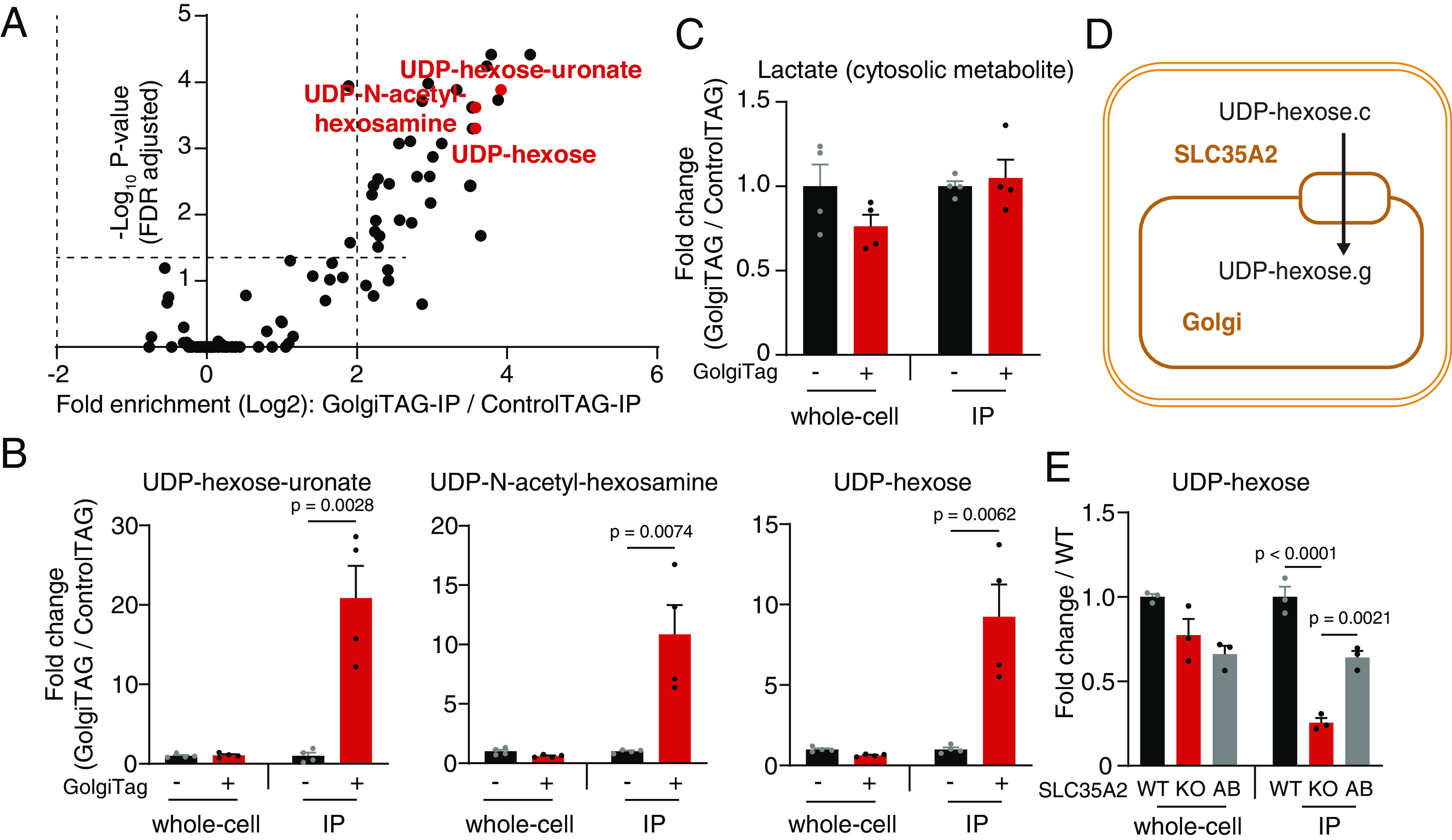
Golgi-IP metabolomics identified UDP-sugars as the main Golgi metabolites and pinpointed substrates for subcellular transporters. (*A*) UDP-hexose-uronate, UDP-N-acetyl-hexosamine, and UDP-hexose are the most enriched species in the Golgi. Volcano plot depicting the fold enrichment of metabolites between IPs derived from ControlTAG and GolgiTAG HEK293 cells (*P*-value corrected by the Benjamini–Hochberg method, FDR = 5%, n = 4). Data available in Dataset S4. (*B*) UDP-hexose-uronate, UDP-N-acetyl-hexosamine, and UDP-hexose are enriched only in the Golgi while the whole-cell level is not affected by the GolgiTAG. Targeted analyses showing fold change in the abundance of UDP species in the IP and whole-cell fractions of GolgiTAG compared to those of ControlTAG cells. (*C*) Lactate, an example of an abundant cytosolic metabolite, is not enriched in GolgiTAG IPs. (*D*) The transporter function of SLC35A2. SLC35A2 transfers UDP-hexose from the cytoplasm to the Golgi. g indicates the Golgi pool and c indicates the cytosolic pool. (*E*) Targeted analysis showing the fold change in the abundance of UDP-hexose in the IP and whole-cell fractions of *SLC35A2* KO and add-back (AB) HEK293 cells compared to those of wild-type (WT) cells. Data are presented as mean ± SEM (n = 4) for *A*–*C* and ± SEM (n = 3) for *E*. Statistical tests: two-tailed unpaired *t* test for *B* and *C* and one-way ANOVA with Tukey’s HSD post-hoc for *E*.

Rapid organelle purification provides an unprecedented opportunity to analyze the labile metabolite content of cellular compartments (10, 11, 16, 17, 41). To determine the metabolite content of the Golgi, we performed untargeted metabolomics using LC/MS on IPs from GolgiTAG or ControlTAG cells. Consistent with their role in the Golgi (42), we found, using unbiased analysis, UDP species to be among the most enriched metabolites in the Golgi (Fig. 4*A* and Dataset S4). Indeed, targeted analyses confirmed that UDP-hexose-uronate, UDP-N-acetyl-hexosamine, and UDP-hexose were enriched 20.9-fold, 10.9-fold, and 9.2-fold in IPs from GolgiTAG cells when compared to those from ControlTAG cells, respectively (Fig. 4*B*). Importantly, whole-cell levels of these species were similar between GolgiTAG and ControlTAG cells (Fig. 4*B*). In contrast to UDP-containing species, we did not observe any enrichment of the abundant cytosolic metabolite lactate in the Golgi samples (Fig. 4*C*), indicating that our method enriches for Golgi specifically.

In addition to UDP-sugars, we identified several metabolites to be enriched in the Golgi (Dataset S4), including phosphocreatine and glutathione (*SI Appendix*, Fig. S5). Phosphocreatine has been implicated in high-energy Golgi signaling involving coupling of creatine kinase and *cis*-Golgi matrix protein GM130 (43). In addition, glutathione is believed to be critical in maintaining the Golgi redox homeostasis (44), and its depletion has been shown to compromise post-Golgi trafficking (45). Another class of metabolites found to be enriched in the Golgi were those related to phosphatidylcholine (PC): phosphocholine and glycerophosphocholine (*SI Appendix*, Fig. S5). As discussed later, PC is the most enriched lipid class in the Golgi. PC is synthesized de novo via the CDP-choline pathway (46), the final step of which involves the conversion of CDP-choline to PC. This reaction is catalyzed by CPT1 and CEPT1, the former of which localizes to the Golgi (47).

The Golgi occupies only a small volume of the whole cell (48); thus, we hypothesized that Golgi-IP coupled with LC/MS can provide a powerful tool to detect subcellular metabolic changes that are missed in whole-cell measurements. To test this, we chose to analyze metabolite changes in *SLC35A2* knock-out (KO) cells, encoding a known transporter that imports UDP-hexose from the cytosol into the Golgi (49) (Fig. 4*D*). Because we could not detect SLC35A2 even in wild-type cells using commercially available antibodies, we verified by mass spectrometry that no detectable SLC35A2 protein was observed in the KO cell GolgiTAG IPs (*SI Appendix*, Fig. S6*A*). We also confirmed that Golgi-IP is as efficient in *SLC35A2* KO cells as in those with wild-type genotype (*SI Appendix*, Fig. S6*B*). Targeted metabolic analysis revealed that UDP-hexose level was significantly reduced in Golgi derived from *SLC35A2* KO cells while its level in the whole cell was unchanged compared to wild-type cells (Fig. 4*E*). Importantly, the level of Golgi-associated UDP-hexose was rescued by reexpressing wild-type SLC35A2 in *SLC35A2* KO cells (Fig. 4*E* and *SI Appendix*, Fig. S6 *C* and *D*). These results provide in vivo evidence for the role of SLC35A2 in transporting UDP-hexose into the Golgi lumen. In addition to UDP-hexose, we surprisingly observed a similar level of reduction for UDP-N-acetyl-hexosamine in the Golgi upon *SLC35A2* ablation, suggesting that SLC35A2 may be able to transport both substrates into the Golgi or that both species are metabolically coupled (*SI Appendix*, Fig. S6*E*). To further exclude the possibility that the biosynthesis of UDP-N-acetyl-hexosamine was inhibited due to the cytosolic accumulation of UDP-hexose upon SLC35A2 ablation, we incubated HEK293T whole-cell lysates with uniformly labeled U-^13^C_6_-glucose with or without unlabeled UDP-hexose supplementation. The amount of de novo synthesized U-^13^C_6_-UDP-N-acetyl-hexosamine was not decreased by UDP-hexose supplementation (*SI Appendix*, Fig. S6*F*).

Taken together, our data show that Golgi-IP metabolomics faithfully uncovers the Golgi metabolome and identifies UDP-hexose and its derivatives as the top Golgi metabolites. In addition, Golgi-IP provides a powerful tool to study Golgi transporters in vivo and uncover subcellular metabolite changes that are difficult to study using whole-cell approaches.

### Lipidomic Analysis of the Golgi Determines its Lipid Content.

**Fig. 5.**
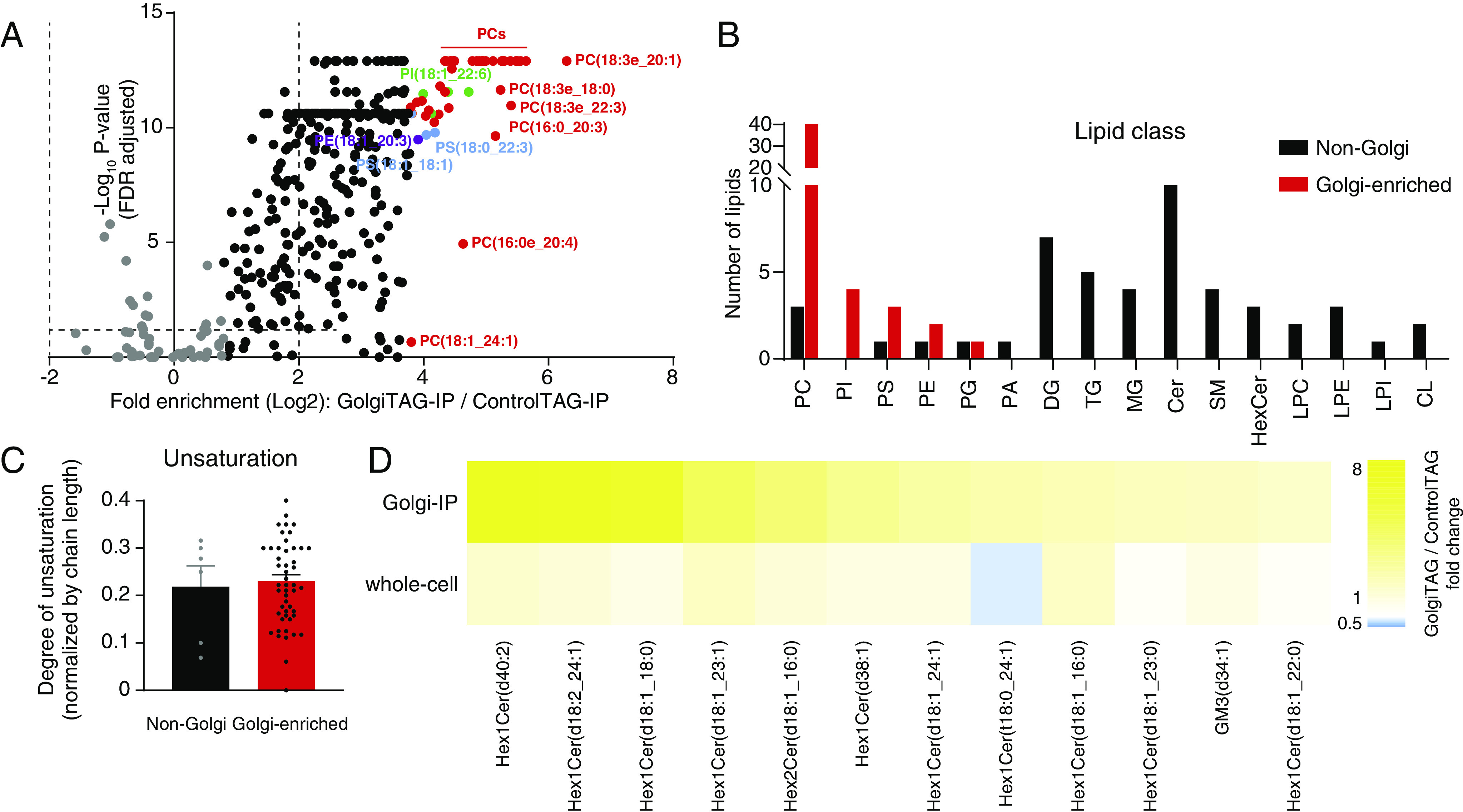
Golgi-IP lipidomics identified phospholipid as the primary Golgi lipid class. (*A*) Volcano plot of the fold enrichment of lipids in IPs derived from GolgiTAG compared to those from control HEK293 cells (*P*-value corrected by the Benjamini–Hochberg method, FDR = 5%, n = 6). Top-50 enriched species are highlighted in multicolors. Non-Golgi lipids are in gray. Data are in Dataset S5. (*B*) Profiling of lipid class in top-50 enriched species in the Golgi against the non-Golgi counterparts. (*C*) Comparison of the degree of unsaturation for phosphatides between the same groups. (*D*) Heatmap showing the fold change of glycosphingolipid abundances in Golgi and whole-cell fractions in GolgiTAG and ControlTAG HEK293 cells. Full lipid names are shown in Dataset S5.

Membrane-bound organelles have a distinct lipid composition that supports their functions (50, 51). To leverage our Golgi-IP to determine the lipid composition of the human Golgi, we performed untargeted lipidomic analysis on GolgiTAG IPs. Unbiased analysis indicated an overall strong enrichment pattern in IPs from GolgiTAG cells compared to those derived from ControlTAG ones (Fig. 5*A* and Dataset S5). We compared the top-50 enriched species in GolgiTAG IPs with those that showed no enrichment, which we define as non-Golgi lipids (at the bottom of the volcano plot) (Fig. 5*B*). The most Golgi-enriched lipids belong to phospholipids, mainly PC followed by phosphatidylinositol (PI) and phosphatidylserine (PS). This finding of PCs and PIs as the most Golgi-enriched lipids is consistent with the reported roles of these lipids in coat protein complex I-mediated retrograde Golgi transport (52). Additionally, reduction of PCs impairs Golgi protein transport (53). To further characterize Golgi-enriched lipids, we also calculated the degree of unsaturation and found that Golgi-enriched lipids are slightly more unsaturated, although the difference was not statistically significant (Fig. 5*C*). In addition to phospholipids, we also observed enrichment of glycosphingolipids including hexosylceramides, dihexosylceramides, and monosialodihexosylgangliosides (Fig. 5*D*). This is consistent with the role of the Golgi in catalyzing the synthesis of glycosphingolipids (54).

In summary, our results show that Golgi-IP can be used to determine the lipid composition of the Golgi and will facilitate future studies to probe its lipid content under various nutrient and disease states.

## Discussion and Conclusions

Subcellular compartments provide optimal environments for specialized biochemical reactions (55). Of these compartments, the Golgi represents a major site for protein maturation and complex lipid biosynthesis. We developed the Golgi-IP method to enable the rapid immunopurification of the intact Golgi. Our method successfully enriches for known Golgi proteins with minimal contamination from other subcellular compartments. Moreover, the Golgi-IP coupled with high-resolution mass spectrometry can be used to perform multimodal analyses of the Golgi molecular content. This has provided comprehensive maps of the human Golgi proteome, metabolome, and lipidome.

Through our proteomic analysis, we confirmed previously known Golgi proteins and identified proteins not previously associated with this organelle. While previously annotated Golgi proteins are mostly associated with the Golgi membrane, the candidates identified by our analysis suggest that certain proteins may be shared between the Golgi and other organelles such as the ER and lysosomes. This may be explained by the anterograde flow of the secretory pathway and the close interaction between the Golgi and these subcellular compartments (56, 57). Our results identify *SLC39A9*-encoded protein ZIP9 as a Golgi-resident protein. ZIP9 is the only known zinc transporter to have a hormone receptor activity (58) and has been described to have an important role in regulating cytosolic zinc levels (59). While its Golgi-association data are limited, a previous report showed a colocalization between ZIP9 and TGN46, a *trans*-Golgi network marker (36). Our proteomic data and immunofluorescence validation support this report and establish ZIP9 as a Golgi-resident protein. TM9SF1, a protein implicated in starvation-induced autophagy, was previously shown to localize to the autophagosome and lysosome (60). We initially speculated that the enrichment of this protein in our GolgiTAG IP was due to the proximity of the Golgi to the lysosome. However, our immunofluorescence analysis confirms that it localizes to the Golgi. KIAA2013 is an uncharacterized protein that is enriched 21-fold in our GolgiTAG IP. Confocal imaging also confirmed it as a Golgi-resident protein. These examples and others provide a proof of principle for the utility of our Golgi-IP method to study the proteomic landscape of the Golgi in health and disease states.

Through untargeted metabolomic analysis of the purified Golgi, we report the metabolite atlas of the human Golgi. Consistent with the Golgi function in protein and lipid glycosylation, we have identified UDP-sugars and their derivatives as the top-enriched polar species in the Golgi. These include UDP-containing species used for the different types of glycosylation reactions including the addition of hexose, N-acetyl-hexosamine, and hexose-uronate. Furthermore, using the Golgi-IP method to analyze the metabolite content of Golgi derived from *SLC35A2* KO cells, we provided subcellular evidence that UDP-hexose is transported by SLC35A2 into the Golgi. Surprisingly, we also found a Golgi-specific reduction in the levels of UDP-N-acetyl-hexosamine, suggesting that SLC35A2 might also transport this metabolite and that UDP-hexose levels in the Golgi are metabolically coupled to those of UDP-N-acetyl-hexosamine. Of importance, none of the observed changes in the levels of these species are detected at the whole-cell level, further indicating the utility of the Golgi-IP method to study the molecular function of subcellular transporters.

Finally, using untargeted lipidomics of purified organelles, we were able to investigate the phospholipid composition of the Golgi. We observed that PCs, PIs, and PSs constitute more than 90% of the top-50 enriched lipids in the Golgi fraction. Consistent with our findings, PC plays a critical role in modulating Golgi protein transport to the plasma membrane in the Chinese hamster ovary cell line MT58 (53). In addition, the enzyme phosphatidylinositol transfer protein β (PITPβ) that exchanges PC and PI at the Golgi–ER interface is essential for Golgi-to-ER retrograde protein transport in HeLa cells (52). In the same work, it was hypothesized that PITPβ is required for importing ER-synthesized PI in exchange for PC in the Golgi, so that the level of PI4P can be maintained for proper Golgi functioning (52). We believe that Golgi-IP holds a great potential in addressing questions related to this biology and exploring the broader area of lipid metabolism in the Golgi.

While insights into organelle biology and metabolism can be achieved through different means including the combined use of multiple mass spectrometry techniques (61), customized isotope tracers (62), and fluorescence sensing (63, 64), direct isolation of organelles via immunoprecipitation has the advantage of allowing the unbiased analyses of their proteomic, metabolomics, and lipidomic content (41). Additionally, the expression of the tags can be easily engineered to allow temporal, spatial, and cell type–specific isolation of organelles in animal models (11, 14, 15). Therefore, we believe that our work that provides a robust technique to isolate and characterize the Golgi will advance the overarching objective to resolve compartmentalized biology with unprecedented subcellular resolution and molecular coverage.

## Materials and Methods

Detailed descriptions of materials and methods are available in *SI Appendix*.

### Generation of GolgiTAG Stable Cell Lines and Golgi-IP Method.

HEK293 cells (The American Type Culture Collection (ATCC). Cat# CRL-1573, RRID:CVCL_0045) were virally transfected with constructs expressing either TMEM115-3×HA (GolgiTAG) or 3×HA (ControlTAG) and positive cells were selected in a puromycin-selection media. Cells stably expressing GolgiTAG or ControlTAG cultured in 15 cm diameter dishes to 100% confluency were homogenized in KPBS using glass Dounce homogenizer. Homogenates were cleared of debris by centrifugation at 1,000 × *g* for 2 min at 4 °C. The supernatant which contains cellular organelles was collected and transferred to a clean 1.5 mL Eppendorf tube containing 100 µL of 25% slurry of anti-HA magnetic bead (Thermo Fisher. Cat# 88837). The mixture was incubated at 4 °C for 5 min ensuring constant gentle shaking. After incubation, the tube containing the mixture was placed on a magnet for 30 s and the supernatant was removed. The beads were washed 3 times with cold KPBS and then resuspended in a solubilization buffer depending on downstream application. Detailed protocols for preparing these stable cell lines and Golgi-IP method were described on protocol.io(10.17504/protocols.io.6qpvrdjrogmk/v1) (10.17504/protocols.io.6qpvrdjrogmk/v1).

### Flow Cytometry Analysis after GolgiTAG Immunoprecipitation.

GolgiTAG or ControlTAG HEK293 cells were treated with either DMSO or 5 µM GolgiTracker for 30 min at 37 °C. The cells were then subjected to Golgi-IP method, as previously described, and resuspended beads were analyzed using a LSRFortessa™ cell analyzer (BD biosciences) with 50,000 events being recorded. Detailed protocol can be found on protocols.io (10.17504/protocols.io.e6nvwk1d2vmk/v1).

### Immunofluorescence Assay.

HeLa cells (ATCC. Cat# CCL-2, RRID:CVCL_0030) grown on 22 × 22 glass coverslips were transiently transfected with appropriate plasmid using polyethylenimine for 24 h. Where indicated, cells were treated with 30 µM nocodazole (Sigma. Cat# M1404) before fixation with paraformaldehyde. The fixed cells were then incubated with primary antibodies for 1 h at room temperature followed by incubation for 1 h with fluorescent-conjugated secondary antibodies and subjected for confocal microscopy. Detailed protocol can be found on protocols.io (10.17504/protocols.io.q26g74qpkgwz/v1).

### Transmission Electron Microscope Sample Preparation.

As described earlier, post Golgi-IP beads were fixed and embedded in Durcupan resin (Sigma. Cat# 44611), and beads were sectioned and contrasted with aqueous uranyl acetate and Reynolds lead citrate, before imaging on a JEOL 1200EX TEM using a SIS III camera. Detailed protocol can be found on protocols.io (10.17504/protocols.io.x54v9y9nqg3e/v1).

### Sample Preparation and LC-MS/MS Analysis for Quantitative Proteomic Analysis.

The GolgiTAG and ControlTAG immunoprecipitated slurry was solubilized in 2% (w/v) (sodium dodecyl sulfate) SDS lysis buffer and tryptic digestion was carried out using S-Tap-assisted On-column tryptic digestion. The digested tryptic peptides were then analyzed on Orbitrap Exploris 480 mass spectrometer using a 50 cm C18 column. The data were acquired in a DIA mode with variable isolation window schemes. Pooled peptide digest was deep fractionated and analyzed on Orbitrap Exploris 480 mass spectrometer in a data-dependent acquisition mode to generate a spectral library. The raw MS data were further processed using the Spectronaut software suite against a Human Uniprot database and search output tables were then processed using Perseus software suite for statistical analysis and data visualization. The detailed protocols have been reported on protocols.io (10.17504/protocols.io.bs3tngnn and 10.17504/protocols.io.kxygxzrokv8j/v1).

### Untargeted Metabolomics.

Polar metabolites were profiled by LC/MS using a pHILIC column and an ID-X tribrid mass spectrometer. Pooled samples were used for data-dependent MS/MS collection. Unbiased differential analysis was performed by Compound Discoverer that integrates both local and online databases. Isotopically labeled amino acids were used for internal standardization. A detailed protocol is described on protocol.io (10.17504/protocols.io.36wgqj3p3vk5/v1 and 10.17504/protocols.io.n2bvj83exgk5/v1).

### Untargeted Lipidomics.

Lipids were profiled by LC/MS using a C18 column and an ID-X tribrid mass spectrometer. Unbiased differential analysis was performed by LipidSearch and Compound Discoverer. Normalization was performed by constant median after blank exclusion. A detailed protocol is described on protocol.io (10.17504/protocols.io.5qpvor3dbv4o/v1 and 10.17504/protocols.io.3byl4jq6jlo5/v1).

## Supplementary Material

Appendix 01 (PDF)

Dataset S01 (XLSX)

Dataset S02 (XLSX)

Dataset S03 (XLSX)

Dataset S04 (XLSX)

Dataset S05 (XLSX)

## Data Availability

Proteomics, lipidomics and metabolomics, R and Python scripts, Raw files for immunoblot, Confocal Immunofluorescence imaging, flow cytometry and Transmission Electron micrograph data have been deposited in ProteomeXchange PRIDE repository (65), MetaboLights (66), and Zenodo (67, 68) (Proteome: PXD038046; Metabolome and Lipidome: identifier number MTBLS6511; 10.5281/zenodo.7347506, 10.5281/zenodo.7656899). Some study data available (All plasmids generated at the MRC Protein Phosphorylation and Ubiquitylation Unit (PPU) at the University of Dundee can be requested through our website https://mrcppureagents.dundee.ac.uk/).
