# Supplementary material for: Golgi-IP, a tool for multimodal analysis of Golgi molecular content
Source: Proc Natl Acad Sci U S A. 2023 May 8;120(20):e2219953120. doi: 10.1073/pnas.2219953120 (PMC10193996; doi:10.1073/pnas.2219953120)
Supplement: Supplementary file 1 — Appendix 01 (PDF) [file pnas.2219953120.sapp.pdf]

Supporting Information for

Golgi-IP, a tool for multimodal analysis of Golgi molecular content

Rotimi Fasimoye[1,2,*], Wentao Dong[2,3,4,5,*], Raja S. Nirujogi[1,2,*], Eshaan S. Rawat[2,3,4,5], Miharu Iguchi[2,3,4,5], Kwamina Nyame[2,3,4,5,6], Toan K. Phung[1,2] , Enrico Bagnoli[1,2], Alan R. Prescott[7], Dario R. Alessi[1,2, #], Monther Abu-Remaileh[2,3,4,5,#]

[1] Medical Research Council (MRC) Protein Phosphorylation and Ubiquitylation Unit, School of Life Sciences, University of Dundee, Dow Street, Dundee DD1 5EH, U.K.

[2] Aligning Science Across Parkinson's (ASAP) Collaborative Research Network, Chevy Chase, MD, 20815, USA.

[3] Department of Chemical Engineering, Stanford University, Stanford, CA 94305, USA.

[4] Department of Genetics, Stanford University, Stanford, CA 94305, USA.

[5] The Institute for Chemistry, Engineering & Medicine for Human Health (ChEM-H), Stanford University, Stanford, CA 94305, USA.

[6] Department of Biochemistry, Stanford University School of Medicine, Stanford, CA 94305, USA.

[7] Dundee Imaging Facility, School of Life Sciences, University of Dundee, Dow Street, Dundee DD1 5EH, U.K.

* Contributed equally.

[#] Corresponding authors: D.R.A.: d.r.alessi@dundee.ac.uk and M.A.-R.: monther@stanford.edu.

This PDF file includes:

Detailed materials and methods

Supplementary figures: S1 to S6

Legends for datasets: S1 to S5

References for SI citations

Materials and Methods

Cell culture

HEK293 cells (ATCC. Cat# CRL-1573, RRID:CVCL_0045), HEK293FT (Invitrogen. Cat# R70007, RRID:CVCL_6911), HeLa (ATCC. Cat# CCL-2, RRID:CVCL_0030), HEK293T wild type (RRID:CVCL_0063) and HEK293T *SLC35A2*-KO (1) were cultured in DMEM (Dulbecco's Modified Eagle Medium, Gibco™) containing 10% (v/v) foetal calf serum, 2 mM L-glutamine, 100 U/ml penicillin and 100 µg/ml streptomycin at 37°C in a humidified incubator maintaining 5% (v/v) CO2. Cells were regularly tested for mycoplasma contamination. Both HEK293T wild type and HEK293T *SLC35A2* knock-out cells were gifts from Mariusz Olczak's lab, University of Wroclaw.

Plasmids

The plasmids (except VSVG and Gag/Pol) used in this study were obtained from the MRC PPU Reagents and Services (https://mrcppureagents.dundee.ac.uk) or other indicated source. Each plasmid was confirmed by sequencing at the MRC Sequencing and Services (https://www.dnaseq.co.uk). Plasmids are available to request via MRC PPU Reagents and Services (https://mrcppureagents.dundee.ac.uk).

Table S1: List of plasmids employed in this study.

Construct	Plasmid Backbone	Source	Catalogue Number
TMEM115-3xHA	pLJC5	MRC PPU Reagents and Services	DU68534
3xHA	pLJC5	MRC PPU Reagents and Services	DU70022

SLC35A2-2xFLAG	pBABED	MRC PPU Reagents and Services	DU72094
KIAA2013-HA	pcDNA5D	MRC PPU Reagents and Services	DU72430
TM9SF1-HA	pcDNA5D	MRC PPU Reagents and Services	DU72445
DIPK1A-HA	pcDNA5D	MRC PPU Reagents and Services	DU72446
C5orf22-HA	pcDNA5D	MRC PPU Reagents and Services	DU72447
SLC39A9-HA	pcDNA5D	MRC PPU Reagents and Services	DU72466
TMEM219-HA	pcDNA5D	MRC PPU Reagents and Services	DU72705
ABHD13-HA	pcDNA5D	MRC PPU Reagents and Services	DU72706

CCDC126-HA	pcDNA5D	MRC PPU Reagents and Services	DU72709
VSVG	Lenti-X HTX	Clontech	631247
Gag/Pol	Lenti-X HTX	Clontech	631247
VSVG	pCMV	Cell Biolabs	RV-110
Gag/Pol	pCMV	Cell Biolabs	RV-111

Transient Transfection

Cell transfection was undertaken using the Polyethylenimine (PEI) method. Except otherwise stated, all cells were transfected at 60% confluency in a 10 cm diameter plate. Briefly, for a 10 cm diameter dish of cells, 6 µg of DNA and 18 µg of PEI were diluted in 0.5 ml of Opti-MEM™ Reduced serum medium (Gibco™), incubated for 30 minutes at room temperature, added dropwise into plates and cells incubated for 24 h at 37°C.

For immunofluorescence transient transfection analysis, 400,000 cells were seeded on ethanol-sterilised coverslip in 6 well 3.5 cm diameter plate containing 22 × 22 mm glass coverslips (VWR. Cat# 631-0125). 2 µg of DNA and 6 µg of PEI were diluted in 0.25 ml of Opti-MEM™ Reduced serum medium (Gibco™), incubated for 30 minutes at room temperature and added dropwise to plates containing cells, which were incubated for 24 h at 37°C.

Buffers

The Triton-X100 cell lysis buffer used in this study to lyse cells and GolgiTAG immunoprecipitates contained 50 mM HEPES-KOH pH7.4, 40 mM NaCl, 2 mM EDTA, 1.5 mM $NaVO_4$, 50 mM NaF, 10 mM NaPyrophosphate, 10 mM Na-β-Glycerophosphate and 1%

(v/v) TritonX-100 and immediately prior to use the buffer was supplemented with cOmplete™ protease inhibitor cocktail (Roche. Cat# 04693116001).

The isotonic buffer used for homogenising cells for the GolgiTAG immunoprecipitation contains potassium-supplemented phosphate saline buffer (KPBS) (136 mM KCL, 10 mM KH_2PO_4. Adjust to pH7.25 with KOH).

The SDS Lysis Buffer used to lyse cells as well as the GolgiTAG immunoprecipitates for proteomics analysis, contained 100 mM HEPES-KOH pH 7.4, 2% (w/v) SDS and immediately prior to use supplemented with cOmplete™protease inhibitor cocktail (Roche. Cat# 04693116001) and PhosStop (Roche. Cat# 4906845001).

The metabolomic solubilization buffer used to resuspend whole-cell lysates and GolgiTAG immunoprecipitates was made of 80% (v/v) Optima LC/MS grade methanol (Fisher. Cat# A456-4) diluted in Optima LC/MS grade water (Fisher, Cat# W6-4). This was supplemented with 500 nM isotopically labelled amino acids (Cambridge Isotope Laboratories. Cat# MSK-A2-S).

The lipidomics solubilization buffer used to resuspend whole-cell lysates and GolgiTAG immunoprecipitates was made of LC/MS grade chloroform (VWR. Cat# 22711.260) and Optima LC/MS grade methanol (Fisher. Cat# A456-4) at a 2:1 (v/v) ratio, supplemented with Splashmix (Avanti. Cat# 330707) (1:1000 dilution).

Cell Lysis for immunoblotting analysis

For cell lysis for immunoblotting analysis, medium is aspirated, cells placed on ice and washed twice with ice cold PBS. For a 10 cm diameter dish, 1 ml Triton-X100 lysis buffer was added, and the cells scraped into 1.5 ml tube, then incubated on ice for 10 minutes. The extract is clarified by centrifugation at 17000 x *g* for 10 minutes at 4°C. Supernatants were collected and stored in aliquots at -80°C and/or used immediately for immunoblotting.

Immunoblotting assay

Whole cells or immunoprecipitated Golgi were lysed in 1% (v/v) Triton-X100 buffer as described above. Protein concentrations of lysates were determined using Pierce™ BCA Protein Assay Kit (Thermo. Cat# 23227) in technical duplicates according to manufacturer's instructions. Lysates were incubated with a quarter of the volume of 4X SDS-PAGE sample buffer [50 mM Tris–HCl, pH 6.8, 2% (w/v) SDS, 10% (v/v) glycerol, 0.02% (w/v) Bromophenol Blue and 1% (v/v) 2-mercaptoethanol] (Novex). For SDS–PAGE, typically 2-20 μg samples were loaded on 4–12% Bis-tris gradient gels (Thermo Fisher Scientific. Cat# WG1402BOX or

Cat# WG1403BOX) and run at 120 V for 120 minutes. Proteins were transferred onto nitrocellulose membranes at 90 V for 90 minutes on ice in transfer buffer [48 mM Tris–HCl, 39 mM glycine and 20% (v/v) methanol]. Transferred membranes were blocked with 5% (w/v) milk in TBST Buffer at room temperature for 60 minutes. Membranes were then incubated with primary antibodies diluted in 5% (w/v) BSA in TBST Buffer overnight at 4°C. After washing in TBST three times, membranes were incubated at room temperature for 1 h with near-infrared fluorescent IRDye antibodies (LI-COR) diluted 1:10,000 in TBST buffer and developed using the LI-COR Odyssey CLx Western Blot imaging system. Detailed protocol is on protocol.io (dx.doi.org/10.17504/protocols.io.bp2l61oxdvqe/v1)

Immunofluorescence assay

The cells indicated in the figure legends were seeded on 22 x 22 mm glass coverslips in 6 well 3.5 cm diameter plates. Transfection was performed as described above for those that were transfected. Where indicated, cells were treated with 30 μM nocodazole (Sigma. Cat# M1404) generated from a 30 mM stock dissolved in DMSO 1 h prior to fixation. For fixation, medium was aspirated, and cells were fixed in 3 ml of 4% (w/v) paraformaldehyde in PBS for 10 minutes at room temperature. Fixed cells were then washed three times at 5 minutes intervals with 0.2% (w/v) bovine serum albumin (BSA) dissolved in PBS. Cells were permeabilized with 1% (v/v) Nonidet P40 diluted in PBS for 10 minutes at room temperature. Permeabilized cells were blocked with 1% (w/v) BSA in PBS for 1 h, then incubated with primary antibody diluted in PBS for 1 h at room temperature in a dark chamber. Antibody dilutions used are described in the table below. This is followed by three washes with 0.2% (w/v) BSA at 5 minutes intervals. Cells were then incubated for 1 h, in a dark chamber, with a mixture of secondary antibodies containing Alexa Fluor 594 donkey anti-Rat (Invitrogen. Cat# A21209. RRID:AB_2535795) and Alexa Fluor 488 donkey anti-rabbit (Invitrogen Cat# A21209. RRID:AB_2535792) or Alexa Fluor 488 anti-mouse (Invitrogen. Cat# A21202. RRID:AB_141607) at 1:500 dilution in PBS. Cells were washed again three times with 0.2% (w/v) BSA, rinsed in MilliQ water, and mounted on glass microscopic slides with Prolong™ Gold antifade reagent with DAPI (Invitrogen. Cat# P36931). Slides were then imaged using Leica TCS SP8 MP Multiphoton Microscope using a 40x oil immersion lens choosing the optimal imaging resolution with 1-pixel size of 63.3 nm × 63.3 nm. Detailed protocol is on protocol.io (dx.doi.org/10.17504/protocols.io.q26g74qpkgwz/v1)

Antibodies for Immunoblotting (IB) and Immunofluorescence (IF).

Table S2: List of primary antibodies employed in this study.

Antibody target	Company	Catalogue number (RRID)	Host species	Dilution for IB	Dilution for IF
ACBD3	Sigma	HPA015594 (RRID:AB_1844491)	Rabbit	1:1000	
ACBD3 (clone 2G2)	Sigma	WH0064746M1 (RRID:AB_2220068)	Mouse		1:1000
GOLGIN97	Cell Signalling Technology	13192 (RRID:AB_2798144)	Rabbit	1:1000	1:100
GM130	Cell Signalling Technology	12480 (RRID:AB_2797933)	Rabbit	1:1000	1:3000
HA	Sigma	11867423001 (RRID:AB_390918)	Rat	1:1000	1:1000
HSP60	Cell Signalling Technology	12165 (RRID:AB_2636980)	Rabbit	1:1000	
Calreticulin	Cell Signalling Technology	12238 (RRID:AB_2688013)	Rabbit	1:1000	

LAMP1	Cell Signalling Technology	3243 (RRID:AB_2134478)	Rabbit	1:1000	
α-Tubulin	Cell Signalling Technology	3873S (RRID:AB_1904178)	Mouse	1:5000	
GCC185 (serum)	Pfeffer's lab		Rabbit		1:500
FLAG	Sigma	F1804 (RRID:AB_262044)	Mouse	1:1000	

Table S3: List of secondary antibodies employed in this study.

**Secondary Antibodies**	**Company**	**Catalogue number (RRID)**	**Dilution for IB**	**Dilution for IF**
IRDye 800CW Donkey anti-Rabbit IgG	LI-COR	926-32213 (RRID:AB_621848)	1:10,000	
IRDye 800CW Donkey anti-Mouse IgG	LI-COR	926-32212 (RRID:AB_621847)	1:10,000	

IRDye 800CW Goat anti-Rat IgG	LICOR	926-32219 (RRID:AB_1850025)	1:10,000	
Alexa Flour 594 Donkey anti-Rat	Invitrogen	A21209 (RRID:AB_2535795)		1:500
Alexa Flour 488 Donkey anti-Rabbit	Invitrogen	A21206 (RRID:AB_2535792)		1:500
Alexa Flour 488 Donkey anti-Mouse	Invitrogen	A21202 (RRID:AB_141607)		1:500

Generation of TMEM115-3xHA (GolgiTAG) stable cell lines

To stably introduce GolgiTAG into the indicated HEK293 and HEK293T cells, we used a lentivirus approach. The first part of the method involves generating lentiviral particles in HEK293FT packaging cells. For this 6 µg lentivirus cDNA construct expressing TMEM115-3xHA (GolgiTAG) was incubated with 3.8 µg pGAG/Pol (Clontech. Cat# 631247) and 2.2 µg pVSVG (Clontech. Cat# 631247) cDNAs, diluted in 0.25 ml Opti-MEM™ Reduced serum medium (Gibco™). This mixture was added to 36 µg of PEI diluted in 0.25 ml Opti-MEM™ Reduced serum medium (Gibco™). After 30 minutes of incubation at room temperature, the mixture was added dropwise into 10 cm diameter dish of 60% confluent HEK293FT cells, which were then incubated for 24 h at 37°C. 24 h post-transfection, medium was removed and replaced with 10 ml of fresh medium and left for another 24 h. This medium, containing lentivirus particles, was collected and used to generate the indicated stable HEK293 or HEK293T cells. These cells, at 60% confluence, were incubated with 5 ml medium containing lentivirus particles diluted with 5 ml fresh DMEM medium in the presence of 10 µg/ml Polybrene (Milipore. Cat# TR1003G) to promote viral infection. After 24 h the medium was replaced with a selection medium supplemented with 2 µg/ml Puromycin (Sigma. Cat# P9620).

The cells that survived and proliferated in 2 μg/ml Puromycin were selected. These cells were permanently cultured in a medium containing 2 μg/ml Puromycin. A Detailed protocol for preparing these stable cell lines is described on protocol.io (dx.doi.org/10.17504/protocols.io.6qpvrdjrogmk/v1)

Introduction of SLC35A2-2xFLAG into HEK293T SLC35A2 knock-out cells

To rescue the expression of SLC35A2 in HEK293T knock-out cells, we utilized a retrovirus approach. 6 μg retrovirus cDNA construct expressing SLC35A2-2xFLAG, was incubated with 3.8 μg pGAG/Pol (Cell Biolabs. Cat# RV-111) and 2.2 μg pVSVG (Cell Biolabs. Cat# RV-110) plasmids, diluted in 0.25 ml Opti-MEM™ Reduced serum medium (Gibco™). This mixture was added to 36 μg of PEI diluted in 0.25 ml Opti-MEM™ Reduced serum medium (Gibco™). After 30 minutes of incubation at room temperature, the mixture was added dropwise into 10 cm diameter dish of 60% confluent HEK293FT cells, which were then incubated for 24 h at 37°C. 24 h post-transfection, medium was removed and replaced with 10 ml of fresh medium and left for another 24 h. This medium, containing lentivirus particles, was collected and used to generate the indicated stable HEK293T cells. These cells, at 60% confluence, were incubated with 5 ml medium containing retrovirus particles diluted with 5 ml fresh DMEM medium in the presence of 10 μg/ml Polybrene (Milipore. Cat# TR1003G) to promote viral infection. After 24 h the medium was replaced with a selection medium supplemented with 500 μg/ml hygromycin (Invivogen. Cat# ant-hg-5). The cells that survived and proliferated in 500 μg/ml hygromycin were selected and were permanently cultured in a medium containing 500 μg/ml hygromycin. A detailed protocol is described on protocol.io (dx.doi.org/10.17504/protocols.io.kqdg3prxpl25/v1)

Golgi-IP Method

Cells cultured on 15 cm diameter dishes were washed twice with cold PBS on ice and incubated with 1 ml ice cold KPBS. Cells were scraped on ice into a 2 ml Eppendorf tube and gently centrifuged at 1000 x *g* for 2 minutes at 4°C to pellet the cells. Supernatant is discarded and pellets are gently resuspended in 1 ml ice cold KPBS. 25 μl of the resuspended cells was taken out and used for “whole-cell fraction” analysis (see below for how this fraction is processed for different applications). The remainder of the suspension was transferred to a 2 ml glass Dounce homogeniser (VWR. Cat# 89026-386) and subjected 25 up and down cycles of homogenization. The extract was centrifuged at 1000 x *g* for 2 minutes at 4°C. The supernatant which contains cellular organelles, was collected, and transferred to a clean 1.5 ml Eppendorf tube containing 100 μl of 25% slurry of anti-HA magnetic beads (Thermo Fisher. Cat# 88837) that had been washed with KPBS Buffer. The resulting mixture was gently

pipetted up and down three times and incubated on a Belly dance shaker (IBI Scientific. Cat# BDRAA115S). After incubation for 5 minutes, the tube was placed on a magnet for 30 sec and the supernatant was removed. Beads were washed 3 times with 1 ml ice cold KPBS. After the last wash, beads were resuspended with a solubilization buffer depending on downstream application. For immunoblotting studies beads were resuspended in 85 µL Triton-X100 lysis buffer for 10 minutes, beads removed, and samples centrifuged at 10, 000 x *g* for 10 minutes and supernatant transferred to a clean 1.5 ml Eppendorf tube and samples stored at -80°C. For proteomic analysis beads were resuspended with 100 µl SDS lysis buffer and incubated for 10 minutes, beads removed, and samples subjected to sonication on a Bioruptor (High intensity 30 sec-ON and 30 sec-OFF of each cycle and a total of 15 cycles were performed), and then centrifuged for 10,000 x *g* for 10 minutes. Samples were transferred to a clean 1.5 ml Eppendorf tube and stored at -80°C. For metabolomic analysis beads were resuspended in 50 µl of metabolomics solubilization buffer and left to incubate at 4°C for 10 minutes. Beads were removed and sample centrifuged for 10,000 x *g* for 10 minutes and transferred to a clean 1.5 ml Eppendorf tube and stored at -80°C. For lipidomics analysis beads are resuspended in 1 ml lipidomics solubilization buffer for 10 minutes at 4°C, beads removed, and the supernatant transferred to a clean 1.5 ml tube. The sample was incubated on a Thermomixer at 1500 rpm for 1 h at 4°C, followed by adding 200 µl 0.9% (w/v) saline (VWR. Cat# L7528) and incubation for further 10 minutes on a Thermomixer at 1500 rpm at 4°C. The mixture was centrifuged at 3000 x *g* for 10 minutes at 4°C to separate the methanol/saline (upper) and chloroform (lower) phases. The lipids are contained in the chloroform phase and the upper phase is removed and discarded. Approximately 600 µl of the lower phase was collected into a fresh 1.5 ml tube and vacuum dried on Speedvac (Thermo Scientific. Cat# SPD140DDA) before storage at -80°C.

Whole-cell fractions were processed in the following ways. For immunoblotting studies, 25 µl of cell suspension in KPBS was resuspended in 125 µl Triton-X100 lysis buffer for 10 minutes, samples centrifuged at 10, 000 x *g* for 10 minutes and supernatant transferred to a clean 1.5 ml Eppendorf tube before storage at -80°C. For proteomic analysis, 25 µl of cell suspension in KPBS was resuspended with 50 µl SDS/lysis buffer, and samples subjected to sonication on a Bioruptor (High intensity 30 sec-ON and 30 sec-OFF of each cycle and a total of 15 cycles were performed), centrifuged for 10,000 x *g* for 10 minutes. Samples were transferred to a clean 1.5 ml Eppendorf tube and stored at -80°C. For metabolomic analysis, 25 µl of cell suspension in KPBS was mixed with 225 µl of metabolomics solubilization buffer and left to incubate at 4°C for 10 minutes. Sample centrifuged at 10,000 x *g* for 10 minutes and transferred to a clean 1.5 ml Eppendorf tube and stored at -80°C. For lipidomics analysis 25 µl of extract was resuspended in 1 ml lipidomics solubilization buffer for 10 minutes on ice and the supernatant transferred to a clean 1.5 ml tube. The sample was incubated on a

Thermomixer at 1500 rpm for 1 h at 4°C, followed by adding 200 µl 0.9% (w/v) saline (VWR. Cat# L7528) and incubated for another 10 minutes on a Thermomixer at 1500 rpm at 4°C. The mixture was centrifuged at 3000 x *g* for 10 minutes at 4°C to separate the methanol/saline (upper) and chloroform (lower) phases. The lipids are contained in the chloroform phase and the upper phase was removed and discarded. Approximately 600 µl of the lower phase was collected (while being careful not to take cell debris on top) into a fresh 1.5 ml tube and vacuum dried on Speedvac (Thermo Scientific. Cat# SPD140DDA) before storage at -80°C.

Detailed protocol for the GolgiTAG anti-HA immunoprecipitation step is on protocol.io (dx.doi.org/10.17504/protocols.io.6qpvrdjrogmk/v1)

Flow cytometry analysis after GolgiTAG immunoprecipitation

Control HEK293 cells or HEK293 cells stably expressing GolgiTAG were grown in 10 cm diameter dishes to 100% confluency. The cells were treated with either DMSO or 5 µM GolgiTracker ((BODIPY™ FL C_5-Ceramide (*N*-(4,4-Difluoro-5,7-Dimethyl-4-Bora-3a,4a-Diaza-*s*-Indacene-3-Pentanoyl)Sphingosine)) (Invitrogen. Cat# D3521) for 30 minutes at 37°C. Media were replaced with fresh media (without GolgiTracker nor DMSO) and cells were incubated for further 30 minutes. Golgi-IP was then performed, and beads washed as described above. The beads were resuspended in 500 µl isotonic buffer (KBPS) in 1.5 ml Eppendorf tube. Three replicates of 4 µl of the resuspended beads were transferred into flow cytometry tubes each containing 400 µl KPBS. Each replicate was analyzed in duplicate using a LSRFortessa™ cell analyser (BD biosciences). 50000 events were recorded and analysed in FlowJo software (RRID:SCR_008520) according to the gating strategy presented in Fig. S1A. Experiment was done in triplicates and data were statistically analyzed using One-Way ANOVA on GraphPad Prism (version 8.3.1) (RRID:SCR_002798). Detailed protocol can be found on protocols.io (dx.doi.org/10.17504/protocols.io.e6nvwk1d2vmk/v1).

Transmission electron microscope sample preparation

Washed magnetic beads from Golgi-IP were fixed in 4% (w/v) paraformaldehyde and 2.5% (v/v) glutaraldehyde diluted in 0.1 M sodium cacodylate buffer (prepared from stock of 0.4 M sodium cacodylate buffer diluted in water and adjusted to pH7.2 with HCl) for 1 h. The beads were washed two times with 0.1 M sodium cacodylate buffer (adjusted to pH7.2 with HCl). For each wash step, beads were separated from supernatant by placing it on a magnet. Beads were then resuspended in 0.1 M sodium cacodylate buffer and centrifuged at 1000 *x g* for 1 minute, to ensure beads are tightly packed. Using a needle, the pellet was gently dislodged and a Pasteur pipette was used to transfer the pellet into a clean glass vial (VWR. Cat# 215-

3571). To ensure rapid dehydration and embedding, the pellet was cut with scalpel into small pieces (about 1 mm^3). These were then post-fixed in 1% (w/v) OsO_4 with 1.5% (w/v) sodium ferricyanide diluted in 0.1M sodium cacodylate buffer for 60 minutes at room temperature. Washed three times in 0.1 M sodium cacodylate buffer (resuspend pellet in buffer, wait 1 minute for pellet to settle and use P1000 pipette to remove supernatant). This was followed by washing with water three times, then beads were incubated with 1% (w/v) tannic acid and 1% (w/v) uranyl acetate for 30 minutes at room temperature. Without further washing, pellets were gradually dehydrated through alcohol series (50%, 70%, 80%, 90%, 95% ethanol) for 10 minutes/series, then into 100% ethanol twice. The beads were then further dehydrated in 100% propylene oxide twice (10 minutes/series), then left overnight in 50% (v/v) propylene oxide and 50% (v/v) Durcupan resin (Sigma. Cat# 44611). Finally, the beads were embedded in 100% Durcupan resin. The resin was polymerised at 60°C for 48 h and sectioned on a Leica UCT ultramicrotome. Sections were contrasted with 3% (v/v) aqueous uranyl acetate and Reynolds lead citrate (1.33g lead citrate, 1.76g sodium citrate; 8 ml 1M NaOH in 50 ml water) before imaging on a JEOL 1200EX TEM using a SIS III camera. Detailed protocol is on protocol.io (dx.doi.org/10.17504/protocols.io.x54v9y9nqg3e/v1)

Sample preparation for Quantitative proteomic analysis

The GolgiTAG immunoprecipitated bead slurry is solubilized in 100 µl of lysis buffer (2% SDS in 100mM HEPES pH8.0, Protease, and phosphatase inhibitor cocktail). The bead slurry was mixed with pipette tips a few times and allowed it to settle on the magnetic rack for 2 minutes. The supernatant was aliquoted into a new 1.5 ml Eppendorf tube and subjected to centrifugation at 17,000 *x g* for 1 minute. The samples were placed on a magnetic rack once again to remove any bead carryover and the supernatant collected into a new 1.5 ml Eppendorf tube. Samples were then subjected to sonication using Bioruptor (High intensity 30 sec-ON and 30 sec-OFF of each cycle and a total of 15 cycles were performed). In parallel, the whole cell fraction was solubilized in 50 µl of SDS lysis buffer and subjected to the Bioruptor assisted sonication and clear lysate was subjected to protein estimation using BCA assay. Both the Immunoprecipitates and whole-cell extracts were processed using S-TRAP assisted trypsin+LysC digestion as described in (2). Briefly, the proteins were reduced by adding 10 mM TCEP and incubated at 60°C for 30 minutes on a Thermo mixer at 1200 rpm agitation. The samples were then brought to room temperature and alkylated by adding 40 mM Iodoacetamide and incubated in dark at room temperature for 30 minutes on a Thermomixer at 1200 rpm agitation. Further, the samples were brought to final 5% (v/vl) SDS and added 1.2% (v/v) phosphoric acid. At this stage, six times the volume of lysate, S-Trap buffer (90% (v/v) methanol in 100mM TEABC) was added and directly loaded on S-Trap micro

columns (Protifi-Co2-micro-80) and centrifuged at 1000 *x g* for 1 minute at room temperature. Columns were further washed by adding 150 µl of S-Trap buffer and centrifuged at 1000 *x g* for 1 minute and this step was repeated another three times. Finally, the S-Trap columns were transferred to new 1.5 ml Eppendorf tubes and supplemented with 1.5 µg of Trypsin+Lys-C in 70 µl and incubated at 47°C for 1.5 h on a Thermomixer followed by the temperature was set to room temperature and left overnight. The peptides were eluted by adding 40 µl of Elution buffer-1 (50mm TEABC) and then 40 µl of Elution buffer-2 (0.1% (v/v) formic acid). Further, 40 µl of Elution buffer-3 (80% (v/v) ACN in 0.1% (v/v) Formic acid) and this step was repeated two more times. The eluates were vacuum dried and solubilized in 60µl of LC-Buffer (3% ACN (v/v) in 0.1% Formic acid (v/v). The peptide amounts were measured using a nanodrop at 224 nm absorbance for an equal loading on LC-MS/MS analysis. The detailed protocol describing STRAP assisted tryptic digestion has been reported on protocols.io (dx.doi.org/10.17504/protocols.io.bs3tngnn).

LC-MS/MS analysis for quantitative proteomics

4 µg of peptide digest was spiked with 1 µl of iRT peptides (Biognsosys). Samples were then transferred into LC glass vials. LC-MS/MS data was acquired on Orbitrap Exploris 480 mass spectrometer which is in-line with Dionex ultimate 3000 nano-liquid chromatography system. Samples were loaded onto a 2 cm pre-column (C18, 5 µm, 100 A°, 100 µ, 2 cm Nano-viper column # 164564, Thermo Scientific) at 5 µl/minute flow rate using loading pump for about 5 minutes and then resolved the peptides on a 50 cm analytical column (C18, 5 µm, 50 cm, 100 A° Easy nano spray column # ES903, Thermo Scientific) at a flow rate of 250 nl/minute flow rate by applying nonlinear gradient of solvent-B (80% (v/v) ACN in 0.1% (v/v) formic acid) for about 125 minutes with a total gradient time and run time of 145 minutes. Data were acquired in DIA-mode with a variable isolation window scheme (The isolation window scheme and key MS parameters are provided in Dataset S3). Full MS was acquired and measured using Orbitrap mass analyzer at 120,000 resolution at m/z 200 in the mass range of 375 - 1500 m/z, AGC target was set at 300% (~ 3E6 ions) with a maximum ion injection time for 30ms. tMS2 (vDIA) scans were acquired and measured using Orbitrap mass analyzer at 30,000 resolution at 200 m/z with an AGC target of 3000% (~ 3E6 ions) with a maximum ion injection accumulation time of 70 ms. Precursor ions were fragmented using normalized higher energy collisional dissociation (HCD) using stepped collision energies of 25, 28 and 32. Both MS1 and MS2 scans were acquired in a profile mode and advanced peak determination algorithm was enabled for accurate monoisotopic envelopes and charge state determination. Loop control was set for 24 scans of tMS2 and one single MS1 scan was acquired per duty cycle. A total of 45 vDIA windows were enabled covering the mass range of 350 to 1500 m/z, the

details of variableDIA isolation window values are provided in Dataset S3. The detailed protocol has been reported in protocols.io (dx.doi.org/10.17504/protocols.io.kxygxzrokv8j/v1)

Spectral library generation and DDA LC-MS/MS analysis

10 µg peptides from Golgi-IP were pooled and subjected to high-pH RPLC fractionation as described in (2). A total of 42 fractions were generated and each fraction was analyzed on Exploris 480 MS platform in a data dependent mode. The LC and MS instrument parameters are provided in Dataset S3. The detailed protocol has been reported in protocols.io (dx.doi.org/10.17504/protocols.io.kxygxzrokv8j/v1)

Proteomics database search and analysis

Pooled GolgiTAG IP DDA data were searched with MaxQuant version (1.6.10.0. URL: https://www.maxquant.org/) (3) against a Uniprot Human database (Release July, 2020 URL: https://ftp.uniprot.org/pub/databases/uniprot/previous_releases/release-2020_04/). The following search parameters were enabled: Trypsin and LysC as a protease with a maximum of two missed cleavages allowed. Deamidation of Asn and Glu, Oxidation of Met and phosphorylation Ser, Thr and Try were set as variable modifications and Carbamidomethylation of Cys was set as a fixed modification. Default search mass tolerances were used i.e. a maximum of 20 ppm MS1 tolerance and 4.5 ppm for main search and 25 ppm for MS2 was allowed. Second peptide and match between runs option was enabled. Data were filtered for 1% FDR at protein, peptide and PSM levels. The protein group file was further annotated to determine the known Golgi annotated Golgi proteins (Compartment.org and Uniprot GO terms) to map the Golgi protein abundance. The abundance rank from high to low were assigned and analysed using Perseus software version (1.6.0.15: URL: https://www.maxquant.org/perseus/) (4). In addition, these data were also searched with the Pulsar search algorithm using Biognosys software suite to construct a hybrid library.
HEK293 cells deep proteome DDA data protein groups table was processed using Perseus software version (1.6.0.15) to estimate the protein copy numbers using Proteomic ruler method (5).

Database search of DIA data and Data analysis

DIA datasets from GolgiTAG-IP, ControlTAG-IP and their whole-cell extracts were imported into the Spectronaut software suite (Version Rubin: 15.7.220308.50606: URL: https://biognosys.com/software/spectronaut/) (6) for library free search or direct DIA to create

a search archive using Pulsar search algorithm. Furthermore, this search archive was appended to the deep Golgi-tag DDA data to create a hybrid library (DDA+DIA) containing 261,484 precursors, 205,320 modified peptides and 9,629 protein groups and this library was used for the main library-based DIA search as depicted in Fig 2A The data were searched against the hybrid library and Human Uniprot database (Released July, 2021. URL: https://ftp.uniprot.org/pub/databases/uniprot/previous_releases/release-2021_02/) using default Spectronaut search settings and filtered for precursor and protein Q-value cut-off of 1%. Excluded single hits and quantification were performed using MS2 area. The protein group tables were exported for further downstream analysis. The output files were further processed to map known Golgi annotated proteins, see our curated list (Dataset S3), known kinases and phosphatases (7, 8), and manually compiled Glycosylation, metabolism and ubiquitylation pathway components. The protein group files were further processed using Perseus software version (1.6.15.0). Missing values were imputed, and data were normalized using quantile normalization. T-test was performed between GolgiTAG-IP and ControlTAG-IP as well as between GolgiTAG-IP and GolgiTAG whole-cell extracts and between GolgiTAG whole-cell and ControlTAG whole-cell conditions and the p-values were corrected using 1% permutation-based FDR. The data visualization was further performed using in-house R scripts, URL: https://www.r-project.org/ (Dataset S3) and figures were edited using Adobe illustrator version 2022: URL: https://www.adobe.com/uk/products/illustrator.html.

Untargeted metabolomics

Profiling of polar metabolites was performed on an ID-X tribrid mass spectrometer (Thermo Fisher Scientific) with an electrospray ionization (ESI) probe. A SeQuant® ZIC®-pHILIC 150 x 2.1 mm column (Millipore Sigma 1504600001) coupled with a 20 x 2.1 mm (Millipore Sigma 1504380001) guard was used to carry out hydrophilic interaction chromatography (HILIC) for metabolite separation prior to mass spectrometry. Mobile phases: A, 20 mM ammonium carbonate and 0.1% ammonium hydroxide dissolved in 100% LC/MS grade water; B, 100% LC/MS grade acetonitrile. Chromatographic gradient: linear decrease from 80-20% B from 0-20 minutes; fast linear increase from 20-80% B from 20-20.5 minutes; 80% B hold from 20.5-29.5 minutes. Flow rate, 0.15 ml/minute. Injection volume, 1.5-2.5 µL. Mass spectrometer parameters: ion transfer tube temperature, 275 °C; vaporizer temperature, 350 °C; Orbitrap resolution, 120,000; RF lens, 40%; maximum injection time, 80 ms; AGC target, $1x10^6$; positive ion voltage, 3000 V; negative ion voltage, 2500 V; Aux gas, 15 units; sheath gas, 40 units; sweep gas, 1 unit. Full scan mode with polarity switching at *m/z* 70-1000 was performed. EASYIC™ was used for internal calibration. For Data-dependent MS2 collection, pooled samples were prepared by combining replicates. HCD collision energies, 15, 30 and 45%;

AGC target, 2x10^6; Orbitrap resolution, 240,000; maximum injection time, 100 ms; isolation window, 1 m/z; intensity threshold, 2x10^4; exclusion duration, 5 seconds; isotope exclusion, enable. Background exclusion was performed via Acquire X with one header blank and the exclusion override factor set to 3 (URL https://www.thermofisher.com/us/en/home/industrial/mass-spectrometry/liquid-chromatography-mass-spectrometry-lc-ms/lc-ms-software/lc-ms-data-acquisition-software/acquirex-intelligent-data-acquisition-workflow.html)

Compound Discoverer (Thermo Fisher Scientific) was used for initial unbiased differential analysis (URL: https://www.thermofisher.com/us/en/home/industrial/mass-spectrometry/liquid-chromatography-mass-spectrometry-lc-ms/lc-ms-software/multi-omics-data-analysis/compound-discoverer-software.html).

In addition to online databases, we also included a local library with both masslist and mzVault spectral archives. Mass tolerance for untargeted discovery, 10 ppm; minimum and maximum precursor mass, 0-5,000 Da; retention time limit, 0-20 min; Peak filter signal to noise ratio, 1.5; retention time alignment maximum shift, 0.5 min; minimum peak intensity, 10,000; compound detection signal to noise ratio, 3. Isotope and adduct settings were kept at default values. Gap filling and background filtering were performed by default settings. Area normalization was performed by constant median after blank exclusion. Compound annotation priority: #1, MassList Search; #2, mzVault Search; #3, mzCloud Search; #4, Predicted Compositions; #5, Chemspider Search; #6, Metabolika Search. The MassList Search was customized with 5 ppm mass tolerance and 1 minute retention time tolerance. The mzVault Search was customized with 10 ppm precursor and fragment mass tolerance and 1 minute retention time tolerance. The mzCloud Search was customized with 10 ppm precursor and fragment mass tolerance. The other searches were performed with default parameters specified in the default workflow "Untargeted Metabolomics with Statistics Detect Unknowns with ID using Online Databases and mzLogic" provided by Compound Discoverer. This untargeted workflow resulted in a total of 3,356 features after the default background exclusion filter. These 3,356 features were further filtered by the following algorithm: 1) MS2 fragmentation spectra were obtained, and 2) at least 1 annotation match in the mzVault, mzCloud or Chemspider Search. To further improve the rigor of our discovery workflow, we performed additional manual filtering based on the following criteria: 1), features with retention time earlier than 3 minutes on this HILIC column, which are nonpolar and should be quantified by a C18 column, were removed, 2) features with predicted compositions containing chemical elements rarely found in human metabolome (e.g. certain halogens) were removed, and 3) features enriched in the Golgi from only one independent experiment were removed. The final filtered list contains 91 compounds

shown in Dataset S4. Rigorous quantification of metabolite abundance was performed by TraceFinder (Thermo Fisher Scientific. https://www.thermofisher.com/us/en/home/industrial/mass-spectrometry/liquid-chromatography-mass-spectrometry-lc-ms/lc-ms-software/lc-ms-data-acquisition-software/tracefinder-software.html) in conjunction with an in-house library of known metabolite standards (MSMLS, Sigma-Aldrich. Curated by LC/MS acquisition on a pHILIC column, June 2022). Isotopically labeled amino acids were used as internal standards. Mass tolerance for extracting ion chromatograms, 5 ppm. A detailed protocol is described on protocol.io (dx.doi.org/10.17504/protocols.io.36wgqj3p3vk5/v1 and dx.doi.org/10.17504/protocols.io.n2bvj83exgk5/v1).

Untargeted lipidomics

Profiling of nonpolar lipids was performed on an ID-X tribrid mass spectrometer (Thermo Fisher Scientific) with a heated electrospray ionization (HESI) probe. An Ascentis Express C18 150 x 2.1 mm column (Millipore Sigma 53825-U) coupled with a 5 x 2.1 mm guard (Sigma-Aldrich 53500-U) was used to carry out C18-based lipid separation prior to mass spectrometry. Mobile phases: A, 10 mM ammonium formate and 0.1% formic acid dissolved in 60% and 40% LC/MS grade water and acetonitrile, respectively; B, 10 mM ammonium formate and 0.1% formic acid dissolved in 90% and 10% LC/MS grade 2-propanol and acetonitrile, respectively. Chromatographic gradient: isocratic elution at 32% B from 0−1.5 minutes; linear increase from 32-45% B from 1.5-4 minutes; linear increase from 45-52% B from 4-5 minutes; linear increase from 52-58% B from 5-8 minutes; linear increase from 58-66% B from 8-11 minutes; linear increase from 66-70% B from 11-14 minutes; linear increase from 70-75% B from 14-18 minutes; linear increase from 75-97% B from 18-21 minutes; hold at 97% B from 21-35 minutes; linear decrease from 97-32% B from 35−35.1 minutes; hold at 32% B from 35.1-40 minutes. Flow rate, 0.26 ml/minutes. Injection volume, 2-4 μL. Column temperature, 55 ◦C. Mass spectrometer parameters: ion transfer tube temperature, 300 ◦C; vaporizer temperature, 375 ˚C; Orbitrap resolution MS1, 120,000, MS2, 30,000; RF lens, 40%; maximum injection time MS1, 50 ms, MS2, 54 ms; AGC target MS1, $4x10^5$, MS2, $5x10^4$; positive ion voltage, 3250 V; negative ion voltage, 3000 V; Aux gas, 10 units; sheath gas, 40 units; sweep gas, 1 unit. HCD fragmentation, stepped 15%, 25%, 35%; data-dependent tandem mass spectrometry (ddMS2) cycle time, 1.5 s; isolation window, 1 m/z; microscans, 1 unit; intensity threshold, 1.0e4; dynamic exclusion time, 2.5 s; isotope exclusion, enable. Full scan mode with ddMS2 at *m/z* 250-1500 was performed. EASYIC™ was used for internal calibration. LipidSearch and Compound Discoverer (Thermo Fisher Scientific. https://www.thermofisher.com/us/en/home/industrial/mass-spectrometry/liquid-

chromatography-mass-spectrometry-lc-ms/lc-ms-software/multi-omics-data-analysis/lipid-search-software.html) were used for unbiased differential analysis. Lipid annotation was acquired from LipidSearch with the precursor tolerance at 5 ppm and product tolerance at 8 ppm. The mass list is then exported and used in Compound Discoverer for improved alignment and quantitation. Mass tolerance, 10 ppm; minimum and maximum precursor mass, 0-5,000 Da; retention time limit, 0.1-30 min; Peak filter signal to noise ratio, 1.5; retention time alignment maximum shift, 1 min; minimum peak intensity, 10,000; compound detection signal to noise ratio, 3. Isotope and adduct settings were kept at default values. Gap filling and background filtering were performed by default settings. The MassList Search was customized with 5 ppm mass tolerance and 1 minute retention time tolerance. Area normalization was performed by constant median after blank exclusion. A detailed protocol is described on protocol.io (dx.doi.org/10.17504/protocols.io.5qpvor3dbv4o/v1 and dx.doi.org/10.17504/protocols.io.3byl4jq6jlo5/v1).

Degree of saturation for phosphatides was normalized by chain length based on the following equation:

Data Availability

Proteomics MS raw data for DDA (Spectral library) and DIA analysis was submitted to ProteomeXchange PRIDE repository (9), the data can be accessed using the dataset identifier, PXD038046. URL: https://www.proteomexchange.org/ R and Python scripts associated with the Proteomics data have been deposited on the Zenodo data repository (10.5281/zenodo.7347506).

Raw files for immunoblot, Confocal Immunofluorescence imaging, flow cytometry and Transmission Electron micrograph, have been deposited on the Zenodo data repository (10.5281/zenodo.7656899). All plasmids generated at the MRC Protein Phosphorylation and Ubiquitylation Unit at the University of Dundee can be requested through our website https://mrcppureagents.dundee.ac.uk/.

Raw MS data files for metabolomics and lipidomics have been submitted to MetaboLights (identifier number MTBLS6511, URL: www.ebi.ac.uk/metabolights/MTBLS6511) (10)

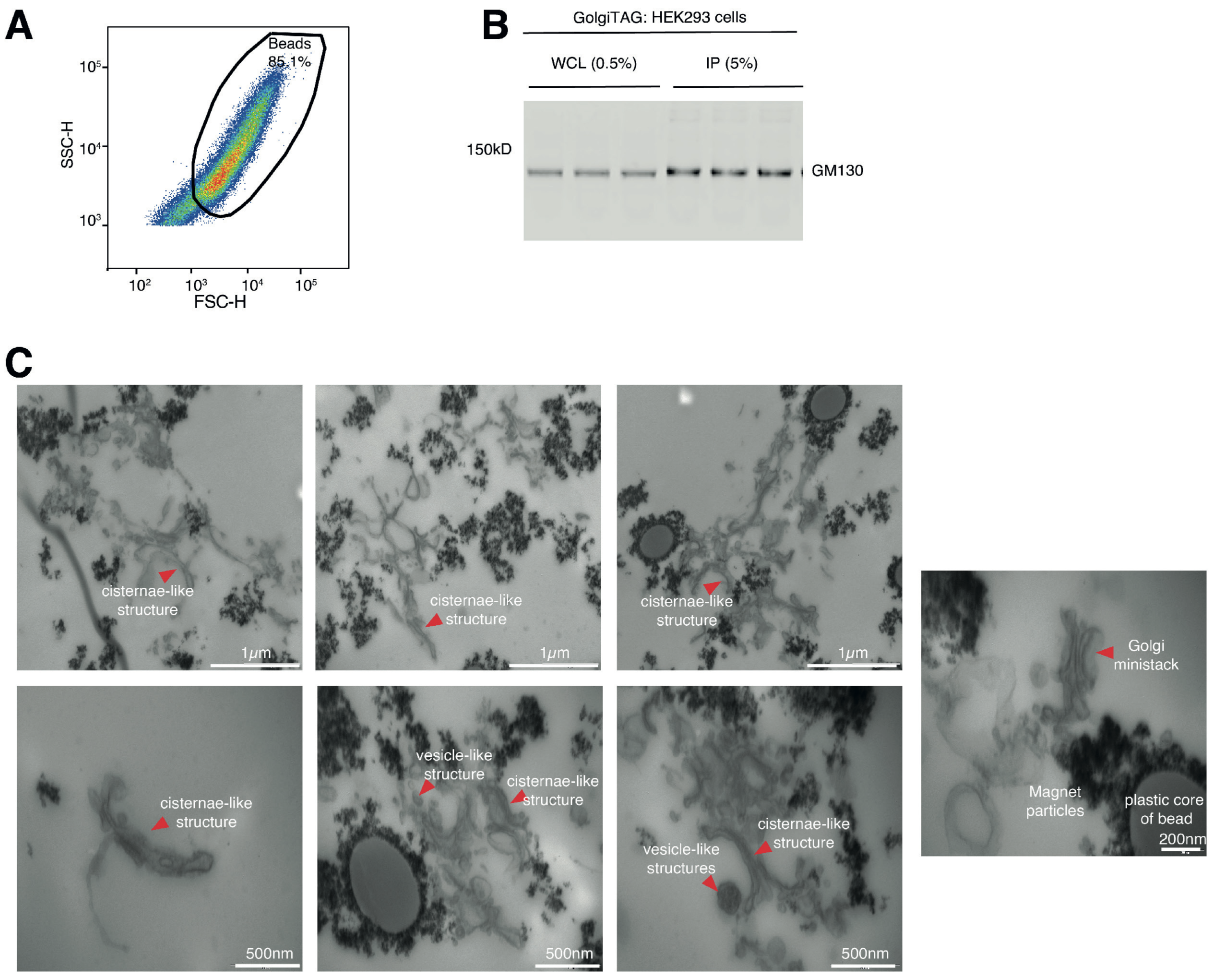

Figure S1: Golgi-IP efficiency and Imaging of Golgi-IP particles. (A) The gating strategy for the beads bound to Golgi for experiment presented in Fig. 1H. The beads bound to Golgi were first gated and selected as indicated, based on complexity (side scatter SSC, Y-axis) and bead size (forward side scatter FSC, X-axis). (B) Immunoblot image used for calculating Golgi-IP efficiency in the experiment presented in Fig. 1J. Lysate from 0.5% total cell as well as 5% resuspended GolgiTAG immunoprecipitates were subjected to immunoblotting by anti-GM130 antibody. (C) Representative Electron Micrograph images of the immunoprecipitated Golgi**.** The Golgi immunoprecipitates were fixed, processed and imaged as described in the materials and methods section and analysed by Transmission Electron Microscopy. Representative images are shown (scale bars are indicated). The Cisternae and vesicle-like structures and intact Golgi ministack are indicated.

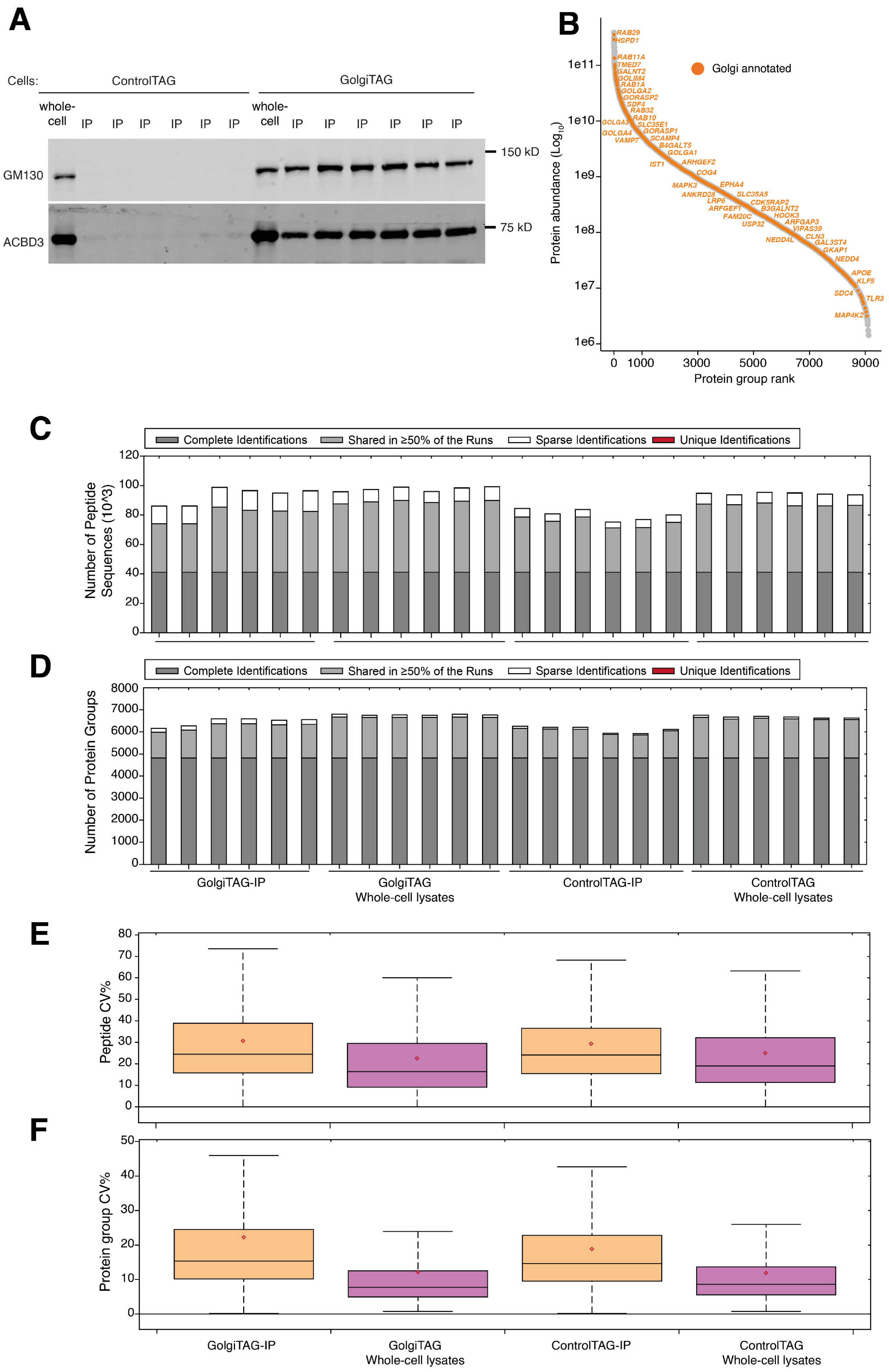
A
Cells:
ControlTAG
GolgiTAG
whole-cell
IP
GM130
ACBD3
150 kD
75 kD
B
Golgi annotated
Protein abundance (Log10)
1e11
1e10
1e9
1e8
1e7
1e6
Protein group rank
0
1000
3000
5000
7000
9000
RAB29
HSPD1
RAB11A
TMED7
GALNT2
GOLIM4
RAB1A
GOLGA2
GORASP2
SDF4
RAB32
RAB10
GOLGA3
SLC35E1
GORASP1
GOLGA4
SCAMP4
VAMP7
B4GALT5
GOLGA1
IST1
ARHGEF2
COG4
MAPK3
EPHA4
ANKRD28
SLC35A5
LRP6
CDK5RAP2
ARFGEF1
B3GALNT2
FAM20C
HOOK3
USP32
ARFGAP3
VIPAS39
CLN3
NEDD4L
GAL3ST4
GKAP1
NEDD4
APOE
KLF5
SDC4
TLR3
MAP4K2
C
Complete Identifications
Shared in ≥50% of the Runs
Sparse Identifications
Unique Identifications
Number of Peptide Sequences (10^3)
0
20
40
60
80
100
120
D
Complete Identifications
Shared in ≥50% of the Runs
Sparse Identifications
Unique Identifications
Number of Protein Groups
0
1000
2000
3000
4000
5000
6000
7000
8000
GolgiTAG-IP
GolgiTAG Whole-cell lysates
ControlTAG-IP
ControlTAG Whole-cell lysates
E
Peptide CV%
0
10
20
30
40
50
60
70
80
F
Protein group CV%
0
10
20
30
40
50
GolgiTAG-IP
GolgiTAG Whole-cell lysates
ControlTAG-IP
ControlTAG Whole-cell lysates

Figure S2: Further analysis of the GolgiTAG immunoprecipitates. (A) Immunoblot analyses of the samples used in Fig. 2B. The whole cell lysates (2 µg) as well as the resuspended GolgiTAG immunoprecipitates (2 µg) were subjected to immunoblotting with the indicated antibodies that are markers for the Golgi. (B) Deep proteomic profiling of pooled GolgiTAG and control immunoprecipitations. Peptides were subjected to bRPLC fractionation, MS data were acquired in DDA mode to generate a spectral library which was further used for DIA data search. Rank abundance plot depicting the protein log intensities on y-axis and protein group abundance rank on x-axis. Known Golgi annotated proteins highlighted in filled circles in orange colour. (C-D) The peptide and protein groups identification summary in each sample from DIA-MS proteomic analysis of GolgiTAG-IP, ControlTAG-IP, and whole-cell lysates (n= 6 in each category). (E-F) Box plots depicting the peptide and protein groups coefficient of variation from DIA-MS proteomic analysis of GolgiTAG-IP, ControlTAG-IP, and whole-cell lysates (n= 6 in each category).

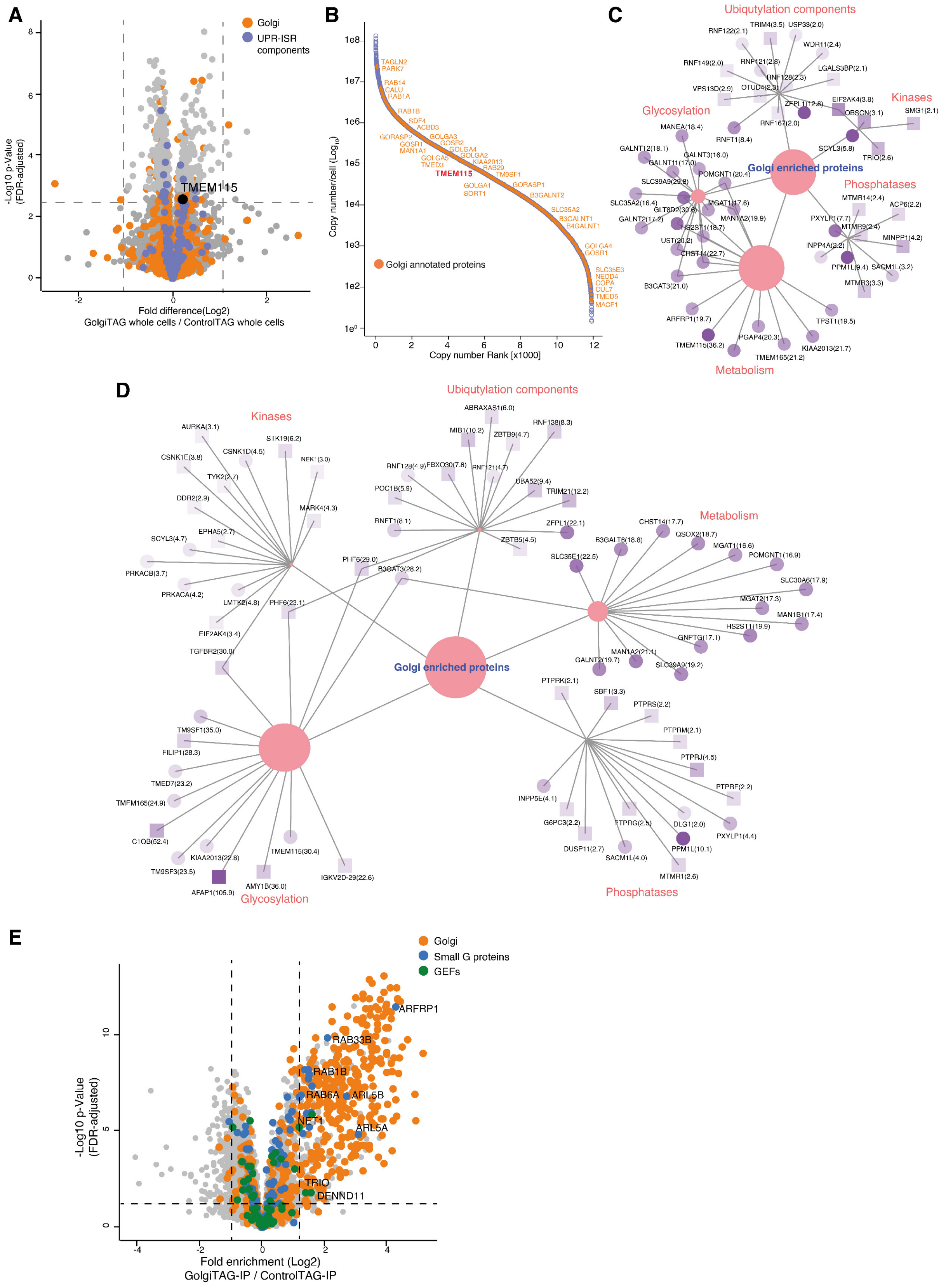

Figure S3: Further proteomic analysis of the GolgiTAG immunoprecipitates. (A) Volcano plot showing the fold difference between the whole-cell lysates from GolgiTAG and ControlTAG HEK293 cells, n= 6 (p-value adjusted for 1% Permutation-based FDR correction,

s0=0.1). The orange dots depict known Golgi annotated proteins curated from databases described in (Table S3) and purple dots depict proteins associated with unfolded protein response and integrated stress response pathway components. (B) Rank abundance plot depicting the protein copy numbers from whole-cell extracts of HEK293 cells. X-axis representing the protein abundance rank (High-Low) and Y-axis representing the estimated copy numbers in log scale. The curated Golgi proteins are shown in filled circles in orange color along with the gene names for selected Golgi proteins (Table S3). (C) Network analysis for enriched proteins in GolgiTAG over control IPs. The proteins enriched in the the GolgiTag IPs versus the control (Table S1) were subjected to cytoscape network analysis (11), using a custom python script (Table S3) and filtered for a fold change value of > 2.0 and 1% FDR for p-value significance. We have selected enzymes involved in ubiquitylation (ligases and deubiqutylases), phosphorylation (kinases and phosphatases) and glycosylation. Proteins previously annotated as Golgi are shown in circles and proteins not previously annotated as Golgi shown in squares. The fold change values are represented as dark (high fold change) to light (low fold) purple color. (D) same as (C) except for those enriched by comparing the GolgiTAG IP to the whole-cell lysates using data from Table S1. (E) Same as Figure 2C, comparison of GolgiTAG over ControlTAG IPs where orange, blue and green dots denoting proteins annotated as Golgi, small G proteins and GEFs respectively.

A

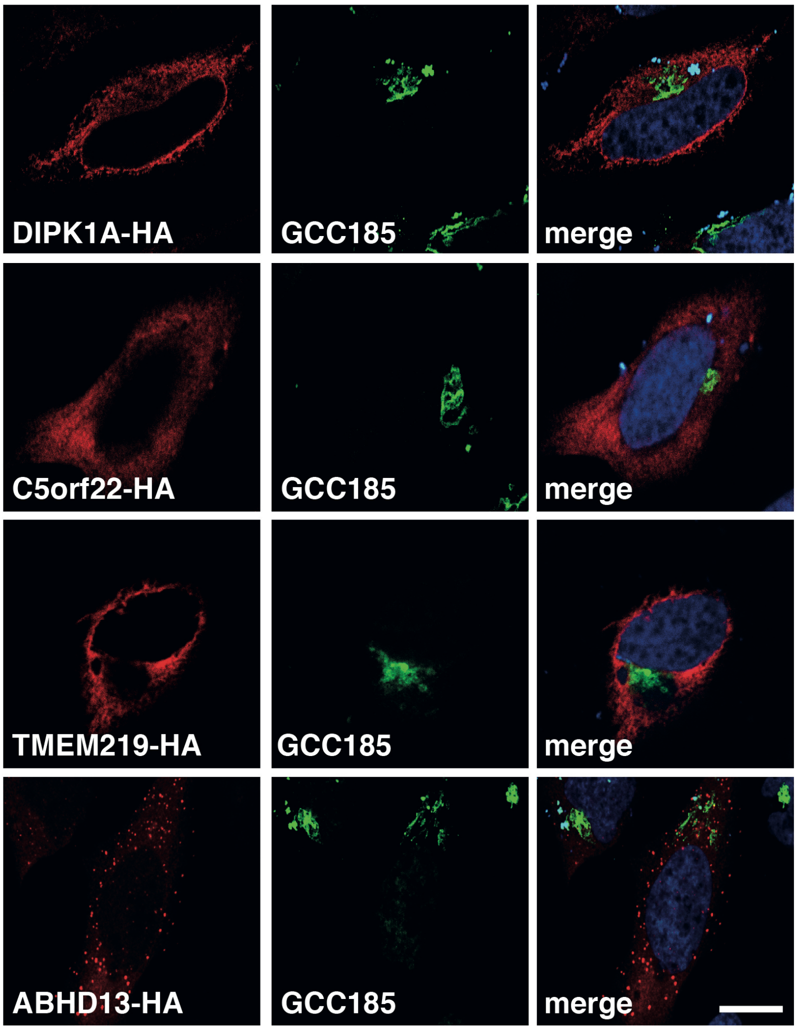

Figure S4: Immunofluorescence analyses of enriched proteins from GolgiTAG IPs that were not previously Golgi annotated. HeLa cells were transiently transfected with plasmids encoding for the expression of the indicated proteins fused to a C-terminal HA tag. 24h post transfection cells were then fixed with 4% (w/v) paraformaldehyde at room temperature and stained with rat anti-HA (red, left panels) and rabbit anti-GCC185 (green, middle panels). The Right panel displays the merged red and green channel. Nuclei were stained with DAPI (blue). Scale bar is 1 μm.

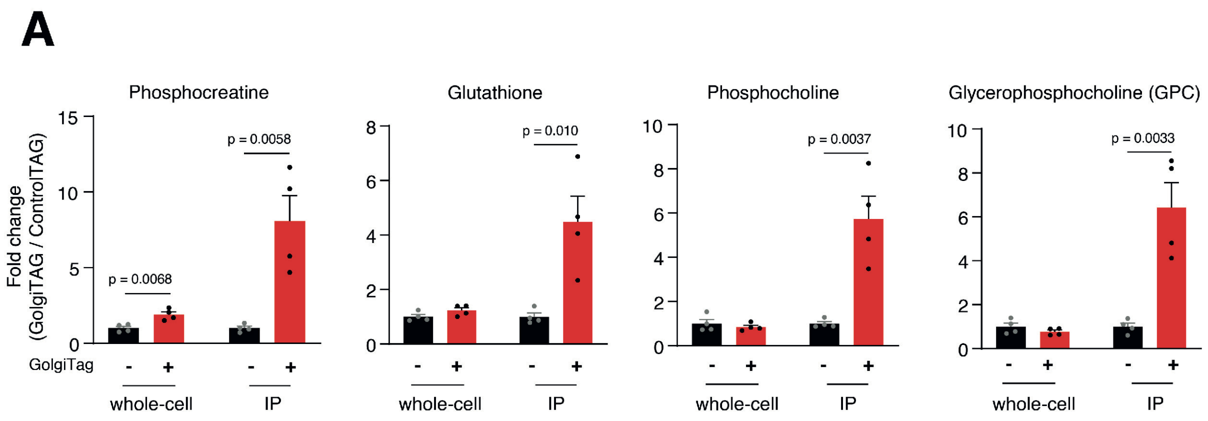

Figure S5: Quantitation of selected Golgi-enriched metabolites. Quantitation of phosphocreatine, glutathione, phosphocholine and glycerophosphocholine (GPC). Fold changes in the abundance of the indicated metabolites in IP and whole-cell fractions of GolgiTAG compared to those of ControlTAG cells are presented as mean ± SEM (n= 4).

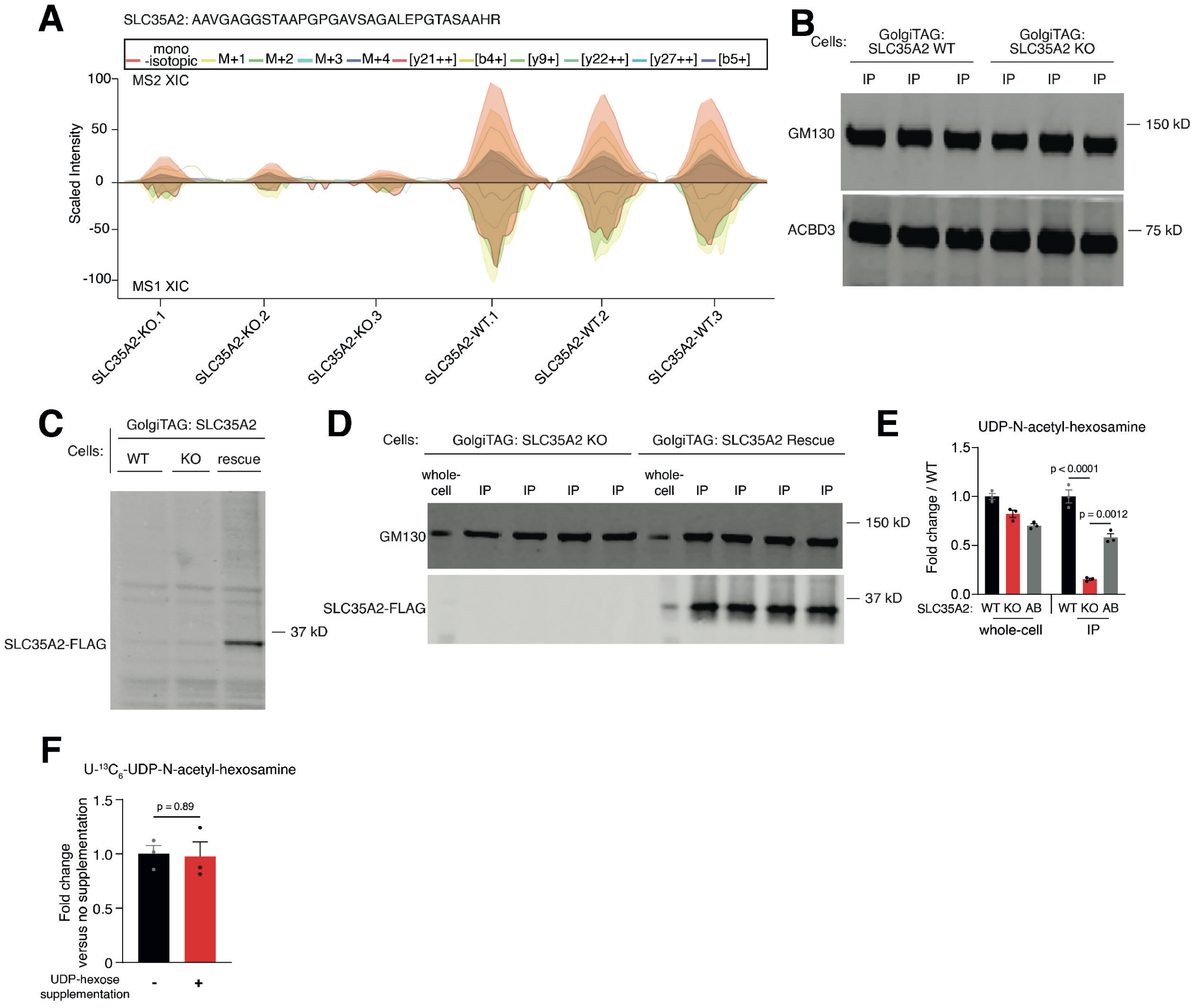

Figure S6: Analyzing *SLC35A2* wild type, knockout and reconstituted HEK293T cell lines. (A to B) Golgi-IP in *SLC35A2* wild-type (WT) and knockout HEK293T cells (n= 3) validate the loss of SLC35A2 protein in the knockout cells**.** (A) The GolgiTAG IPs were analyzed by DIA mass spectrometry and the extracted ion chromatography (XIC) of an SLC35A2 selective peptide (AAVGAGGSCAAPGPGAVSAGALEPGTASAAHR) which displayed robust intensity in wild-type cells while lost in the knockout cells is presented. (B) Immunoblot analyses of the GolgiTAG IP from the indicated cell lines. 2 µg lysates were subjected to immunoblotting with the indicated antibodies that are markers for the Golgi. (C) Wild-type, *SLC35A2* knockout and *SLC35A2* knockout HEK293T cells reconstituted with wild-type SCL35A2 fused to the C-terminal Flag tag (rescue) all stably expressing the GolgiTAG were lysed in a buffer containing 1% (v/v) Triton-X100 and subjected to immunoblot analysis with the Flag antibody. (D) SLC35A2 knockout and rescued HEK293T cells were subjected to Golgi-IP. The whole cell lysates (2 µg) as well as the resuspended GolgiTAG IP (2 µg) were analyzed by immunoblotting with the indicated antibodies. (E) Targeted analysis showing fold change in the abundance of UDP-N-acetyl-hexosamine in the IP and whole-cell fractions of

SLC35A2 KO and rescued (AB) HEK293T cells compared to those from wild-type cells. Data are presented as mean ± SEM (n= 3). Statistical tests: two-tailed unpaired t-test. (F) Quantitation of uniformly labeled U-13C6-UDP-N-acetyl-hexosamine with or without supplementation of unlabeled UDP-hexose in whole-cell lysates from HEK293T cells. Data are presented as mean ± SEM (n= 3).

Dataset S1: DIA-based quantitative proteomic analysis of immunoprecipitates and whole-cell lysates derived from GolgiTAG and ControlTAG HEK293 cells. We have applied a stringent 1% permutation-based FDR and 2-fold cut-off to consider a protein enriched Golgi protein in the current analysis. We also list potential secreted proteins in our enriched Golgi annotated protein data set using Signal-P 6.0 prediction algorithm (31). Out of the 115 proteins that are predicted to contain signal peptide for Golgi localisation, 83 proteins are identified as Golgi proteins that were clearly enriched, and the remaining 32 proteins were identified but not enriched. The presence of a Signal peptide for Golgi localisation is indicated.

Dataset S2: Pooled GolgiTAG-IP Data dependent Acquisition (DDA)-based MS analysis used to generate Spectral library for DIA.

Dataset S3: List of manually curated Golgi proteins list. We include information pertaining to each Golgi protein as 3 categories namely, detected-enriched, detected-not enriched and not detected in HEK293 cells.

Dataset S4: Untargeted metabolomics of Golgi derived from GolgiTAG and ControlTAG HEK293 cells. We included all features detected by untargeted LC/MS. 2) P-values were calculated by ANOVA with Tukey HSD test. The p-values were corrected by the Benjamini-Hochberg method, FDR = 5%. N = 4 per line.

Dataset S5: Untargeted lipidomics of Golgi derived from GolgiTAG and ControlTAG HEK293 cells. We included all lipids detected by untargeted LC/MS. P-values were calculated by ANOVA with Tukey HSD test. The p-values were corrected by the Benjamini-Hochberg method, FDR = 5%. N = 6 per line.

Supporting References

1. B. Szulc *et al.*, Biosynthesis of GlcNAc-rich N- and O-glycans in the Golgi apparatus does not require the nucleotide sugar transporter SLC35A3. *J Biol Chem* **295**, 16445-16463 (2020).
2. R. S. Nirujogi *et al.*, Development of a multiplexed targeted mass spectrometry assay for LRRK2-phosphorylated Rabs and Ser910/Ser935 biomarker sites. *Biochem J* **478**, 299-326 (2021).
3. J. Cox, M. Mann, MaxQuant enables high peptide identification rates, individualized p.p.b.-range mass accuracies and proteome-wide protein quantification. *Nat Biotechnol* **26**, 1367-1372 (2008).
4. S. Tyanova *et al.*, The Perseus computational platform for comprehensive analysis of (prote)omics data. *Nat Methods* **13**, 731-740 (2016).
5. J. R. Wisniewski, M. Y. Hein, J. Cox, M. Mann, A "proteomic ruler" for protein copy number and concentration estimation without spike-in standards. *Mol Cell Proteomics* **13**, 3497-3506 (2014).
6. R. Bruderer *et al.*, Extending the limits of quantitative proteome profiling with data-independent acquisition and application to acetaminophen-treated three-dimensional liver microtissues. *Mol Cell Proteomics* **14**, 1400-1410 (2015).
7. G. Manning, D. B. Whyte, R. Martinez, T. Hunter, S. Sudarsanam, The protein kinase complement of the human genome. *Science* **298**, 1912-1934 (2002).
8. M. J. Chen, J. E. Dixon, G. Manning, Genomics and evolution of protein phosphatases. *Sci Signal* **10** (2017).
9. E. W. Deutsch *et al.*, The ProteomeXchange consortium in 2017: supporting the cultural change in proteomics public data deposition. *Nucleic Acids Res* **45**, D1100-D1106 (2017).
10. K. Haug *et al.*, MetaboLights: a resource evolving in response to the needs of its scientific community. *Nucleic Acids Res* **48**, D440-D444 (2020).
11. P. Shannon *et al.*, Cytoscape: A software environment for integrated models of biomolecular interaction networks. Genome Res. **13**, 2498-2504 (2003).
